# Application of In Silico Filtering and Isothermal Titration Calorimetry for the Discovery of Small Molecule Inhibitors of MDM2

**DOI:** 10.3390/ph15060752

**Published:** 2022-06-16

**Authors:** Hen Alali, Itai Bloch, Irena Rapaport, Luisa Rodrigues, Inbal Sher, Tamar Ansbacher, Maayan Gal

**Affiliations:** 1 Department of Oral Biology, Goldschleger School of Dental Medicine, Faculty of Medicine, Tel Aviv University, Tel Aviv 6997801, Israel; henalali@mail.tau.ac.il (H.A.); luisarodrigues.10@gmail.com (L.R.); inbalasher87@gmail.com (I.S.); tamar.ansbacher@mail.huji.ac.il (T.A.); 2 Migal—Galilee Research Institute, Tel-Hai Academic College, Upper Galilee 12210, Israel; itaib@migal.org.il (I.B.); irenar@migal.org.il (I.R.); 3 Hadassah Academic College, Jerusalem 91010, Israel

**Keywords:** MDM2, isothermal calorimetry, drug discovery, virtual screening, de novo synthesis, protein–protein interaction inhibitors

## Abstract

The initial discovery phase of protein modulators, which consists of filtering molecular libraries and in vitro direct binding validation, is central in drug discovery. Thus, virtual screening of large molecular libraries, together with the evaluation of binding affinity by isothermal calorimetry, generates an efficient experimental setup. Herein, we applied virtual screening for discovering small molecule inhibitors of MDM2, a major negative regulator of the tumor suppressor p53, and thus a promising therapeutic target. A library of 20 million small molecules was screened against an averaged model derived from multiple structural conformations of MDM2 based on published structures. Selected molecules originating from the computational filtering were tested in vitro for their direct binding to MDM2 via isothermal titration calorimetry. Three new molecules, representing distinct chemical scaffolds, showed binding to MDM2. These were further evaluated by exploring structure-similar chemical analogues. Two scaffolds were further evaluated by de novo synthesis of molecules derived from the initial molecules that bound MDM2, one with a central oxoazetidine acetamide and one with benzene sulfonamide. Several molecules derived from these scaffolds increased wild-type p53 activity in MCF7 cancer cells. These set a basis for further chemical optimization and the development of new chemical entities as anticancer drugs.

## 1. Introduction

Interfering with protein–protein interactions (PPIs) is a sought-for aim in drug discovery [1,2,3]. Indeed, the large number of PPIs and their relevance to diseases marks them as promising therapeutic targets [4,5,6]. The interaction surface of the murine double minute 2 (MDM2) with the tumor suppressor p53 is a well-established drug target [7,8,9,10]. The transcription factor p53 is among the foremost proteins for maintaining balanced cellular regulation. Following cellular stress signals, activated p53 is key to regulating genes responsible for cell cycle arrest and apoptosis [11,12,13,14]. The central role of p53 in eliminating cellular defects that otherwise advance tumor formation and cancer denotes it as an imperative cellular regulator. Moreover, about half of human cancers are related to inactivity of p53 due to deletions or mutations in the TP53 gene encoding the protein, leading to p53 loss of activity [15,16]. However, high occurrences of cancers in which wild-type p53 retains its activity have also been characterized. Such cancers are mainly related to the interaction of p53 with its negative regulators, among these the E3-ligase MDM2, which ubiquitinates p53 and promotes its proteasomal degradation via the ubiquitin–proteasome system [17,18,19]. This poses MDM2–p53 PPI as an attractive therapeutic target, due to its direct linkage with cancer. Indeed, a large number of modulators, particularly in the form of small organic molecules that bind MDM2 and interfere with its binding to p53, have been discovered; several have reached clinical trials [9,20,21,22,23].

Roughly speaking, the discovery of new compounds capable of binding a defined epitope on a target protein is based on the filtering of pre-existing molecular libraries. This can be pursued through in vitro high-throughput screening or virtual screening. The application of high-throughput screening has resulted in the discovery of a number of molecules, including the first MDM2 inhibitor Nutlin-3 [24,25]. In addition, the relatively high body of available structural data of MDM2 inhibitors in complex with MDM2 has accelerated the discovery of new chemical entities by computational approaches [26,27,28,29,30,31,32,33]. Regardless of the screening approach, due to the hydrophobic characteristic of the MDM2–p53 interaction surface, most molecules are naturally lipophilic; and a water-exposable hydrophilic site was shown to be required to maximize binding affinity [34]. Indeed, seemingly minor variations between some of the most successful compounds such as Nutlin-1 and Nutlin-3, or WK23 and WW298, confirm that these considerations are essential for an optimized active molecule [24,35]. Following this rationale and relying on the diverse structural data, the current work involved in silico screening of small molecules based on an ensemble of MDM2 structures. The latter were validated for binding to MDM2 and evaluated for their ability to increase p53 activity in MCF7 cancer cells.

## 2. Results

### 2.1. In Silico Screening of a Virtual Small Molecule Library

**Figure 1:**
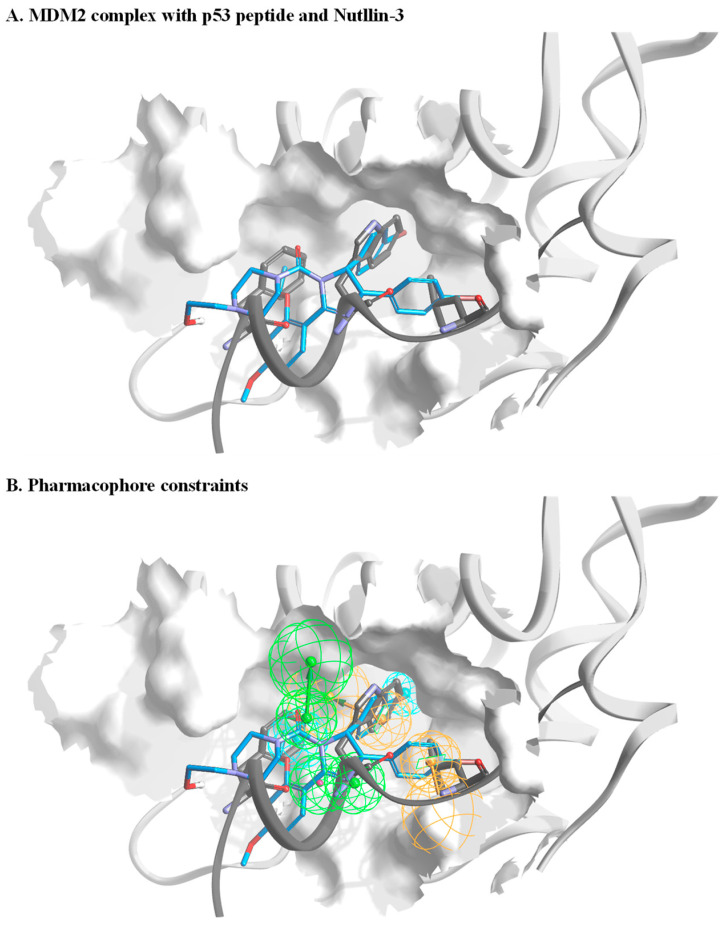
Setting pharmacophore constraints. (**A**) A general overview of MDM2 in a complex with p53 peptide (dark grey) and Nutlin-3 (blue). The structures are based on PDBs, 3DAB, 4DIJ, 3LNZ, 1YCR, and 1RV1. The peptide backbone is shown with only side chains of the p53 peptide that are in direct interaction with MDM2. (**B**) An illustration of pharmacophore constraints that were defined based on analysis of the MDM2 structures. The green spheres represent the location and orientation of potential acceptors within the bound ligand, orange spheres represent the location and direction of the aromatic system, and cyan represents the location of a hydrophobic part.

To filter molecules with the ability to bind MDM2, we performed virtual screening of a small molecule library against MDM2. Synthetic chemistry, particularly of new chemical entities, is often a bottleneck in the initial phase of drug discovery. Thus, we profiled a virtual library based on 20 million commercially available small molecules collected from chemical catalogs. This step ensured that the filtering process would yield a selected set of molecules for in vitro characterization. Our in silico screening consisted of several steps. In the first step, all available MDM2 structures and co-crystal structures were analyzed. These data yielded the pharmacophore constraints for filtering the molecules. Figure 1A illustrates the structural model of the MDM2 complex with p53-derived peptide and Nutlin-3. Based on such a model and together with additional co-complexes, constraints for a representative pharmacophore were defined (Figure 1B). We then performed a crude filtering step of the initial molecules, which resulted in a profiled library of ca. 500,000 molecules. This library was docked onto an MDM2 structure model. An additional aspect in our approach is that docking was performed against an averaged MDM2 structure, which was calculated from an ensemble of MDM2 published structures (PDBs 3DAB, 4DIJ, 3LNZ, 1YCR, and 1RV1) [24,36,37,38,39]. This ensured that our selected set of molecules, which originated from docking, would cover a diverse chemical space. Moreover, they would have the potential to bind the protein in the solution in which it is inherently dynamic and naturally resides in different conformations. In the last step, the highest-ranked molecules were manually selected, and a focused set of ca. 130 small molecules was purchased for in vitro validation. A criterion of selection of the final list of tested compounds was that each one represented a unique chemical genus. This was to ensure the broadest possible chemical space was covered. Figure S1 illustrates the in silico screening workflow until the selection of the final virtual hits.

### 2.2. Secondary In Vitro Screening by Isothermal Calorimetry (ITC)

**Figure 2:**
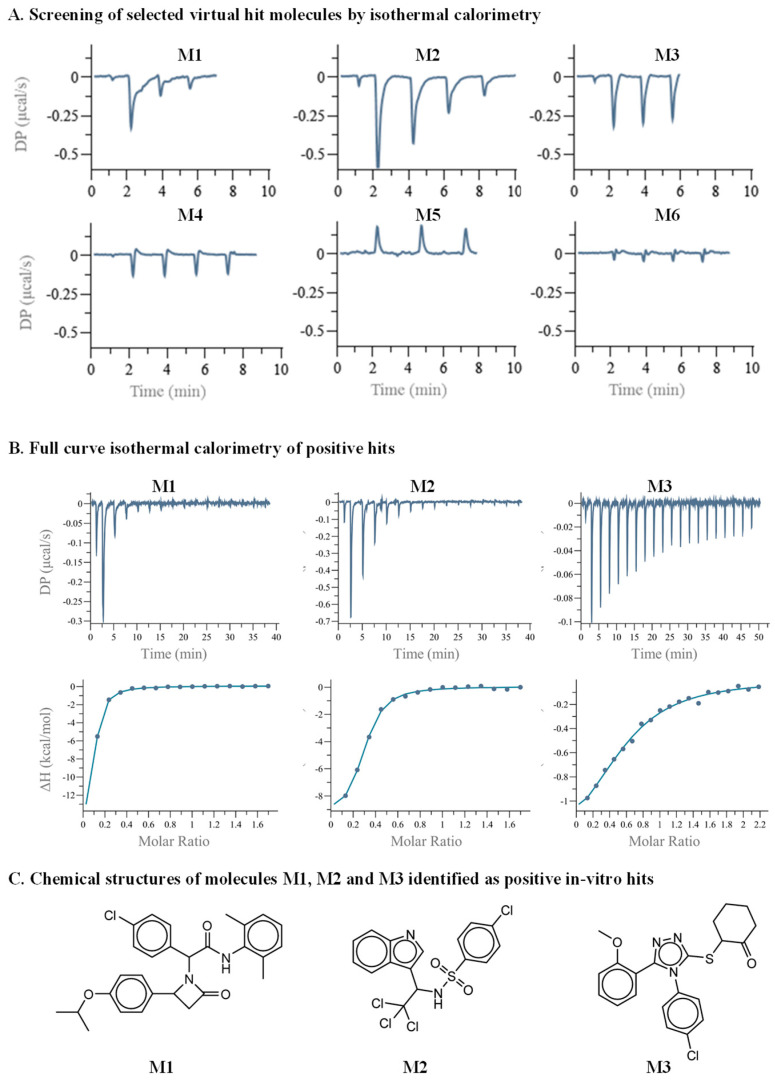
Secondary in vitro screening by ITC. (**A**) Three or four consecutive injections were executed, of MDM2 in the ITC syringe, at a concentration of 300 µM, to the small molecules in the ITC cell, at a concentration of 30 µM. The top row shows binding curves of molecules M1 to M3. The bottom row shows nonbinding curves of molecules M4 to M6. (**B**) Full ITC titration curves. (**C**) Chemical structures of molecules M1, M2, and M3.

Preliminary hits originating from computational prediction and filtering likely have relatively low binding affinity towards the target protein. It is thus imperative to test the direct binding of the selected hits using a robust and sensitive method to minimize both false negative and false positive readouts of screening hits. Among the various biophysical tools, ITC is a gold-standard technique capable of evaluating the direct binding of a molecule to its target protein. ITC is a label-free technique and has the ability to measure a relatively broad range of dissociation constant (k_d_) values and to deliver the thermodynamic parameters of the binding entropy (ΔS) and enthalpy (ΔH) [40,41]. The main drawbacks of using ITC as a screening tool are the low throughput and the large amount of required material. The latter is of immense importance when seeking low binding affinities, as the concentration of the molecules in the ITC cell and in the syringe should be in the order of ten- and one-hundred-fold the k_d_, respectively. This necessitates a large amount of protein. An additional hurdle is that small drug-like molecules are relatively hydrophobic, often reaching a solubility limit of several tens of micromolars in water-based buffers. To partially mitigate these limitations, we employed a unique screening protocol, in which the small molecule is placed in the ITC cell at a concentration of 30 µM, and MDM2 is in the syringe at a concentration of 300 µM. Moreover, initial screening is executed by three consecutive injections, and a binding decision is obtained by manual inspection or in a semi-automated fashion based on the thermogram magnitude and pattern [42,43]. The six panels of Figure 2A illustrate the raw data originating from such screening. The bottom row shows three nonbinding molecules (M4–M6) in which the differential power (DP) values of the experiment were either relatively low or without change during the various injections. The top row shows panels in which both the total magnitude and pattern suggest that binding has occurred. To further validate the screening results, we measured a full titration curve for each positive hit. This employed a complete series of injections of MDM2 to each molecule in the ITC cell. Figure 2B shows the titration curves that were fitted to a single-site binding model. The compounds M1, M2, and M3 yielded affinity values of 2.85, 2.08, and 16.6 µM, respectively. Importantly, the DP values for M1 and M2 did not yield plateaus, suggesting that the binding was relatively weak. Figure 2C shows the chemical structure of the three candidates that were identified as MDM2 binders.

### 2.3. Binding Evaluation of Structure-Similar Chemical Analogues

**Figure 3:**
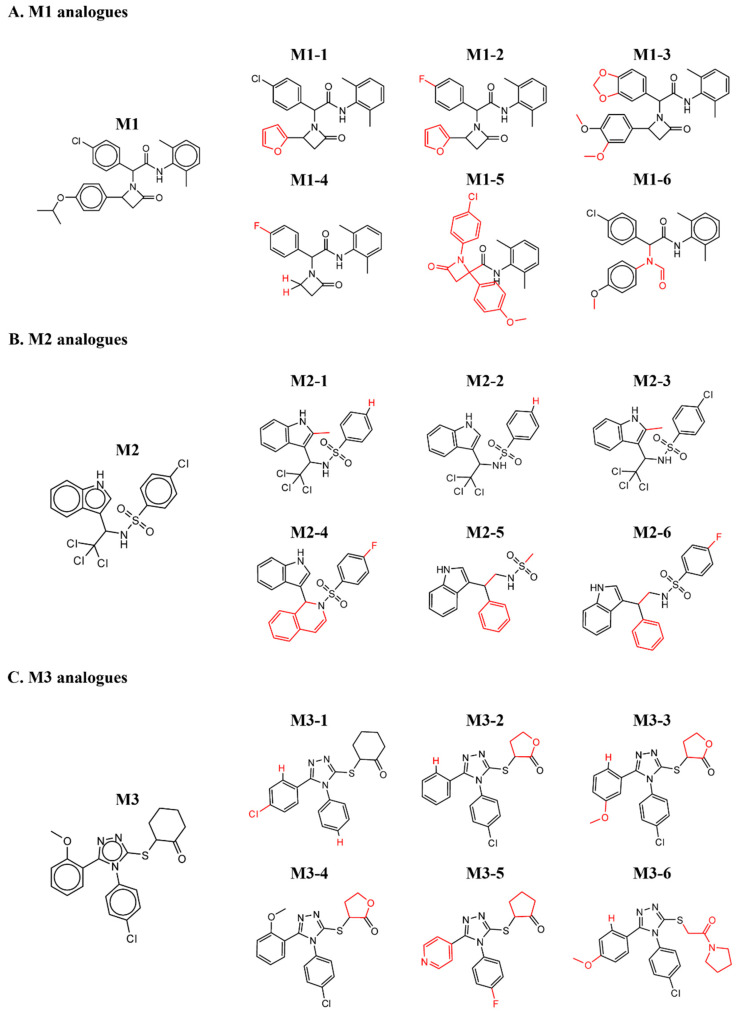
Chemical structures of analogues that were tested for binding to MDM2. The red color shows the substitutions that differ from the original hit.

A major challenge of protein–drug interaction screening protocols, and particularly of preliminary hits of PPI inhibitors, is the relatively high rate of false positive hits. Among the various approaches to eliminating false readouts, studying available off-the-shelf structure-similar chemical analogues of the initial hit is efficient and cost-effective. Such derivatives, which hold a low number of chemical changes compared to the original hit, are expected to retain, at least to some extent, binding to the target protein, while presenting a range of binding affinities. However, if the preliminary hit is a singular point within its proximate chemical space, it may represent an artifact that arises due to an experimental error or sample impurities. Figure 3A shows the M1 structure-similar analogues that were tested. The functional groups that differed from the original M1 structure are highlighted in red. Apart from M1-5 and M1-6, the other analogues retained the (2,6-dimethylphenyl)-2-(2-oxoazetidin-1-yl)-acetamide, and the additional two benzene groups in the molecule were sampled. M1-2 to M1-5 did not preserve binding to MDM2. While the M1-6 molecule did not preserve oxoazetidine, both M1-1 and M1-6 showed low binding affinity towards MDM2. Figure S2 shows the ITC curves that resulted from titrating MDM2 into the M1 derivatives. Figure 3B shows the M2 analogues that were tested. Here we sampled the chemical groups on both sides of the central methanesulfonamide. Of note, compounds containing sulfonamide were recently reported as showing promising activity towards MDM2 [44]. M2-2 that has a benzene instead of the chlorobenzene, and M2-3 that has an additional 2-methyl on the indole, retained binding towards MDM2. However, no binding was detected in any of the other chemical structures that were tested. Figure S3 shows the ITC curves that originated from the titration of MDM2 to each of the M2 analogues. Figure 3C shows the M3 analogues that were tested. All three functional groups around the triazole were sampled. The methoxybenzene group was alternated with various benzene derivatives, the chlorobenzene group with benzene and fluorobenzene, and the cyclohexanone group with tetrahydrofuran derivatives. None of the structure-similar chemical analogues of M3 showed binding towards MDM2. Figure S4 shows the ITC curves of the explored derivatives. Although the low number of structures sampled limits the physiochemical understanding of the interaction, the binding of additional chemical analogues of molecules M1 and M2 to MDM2 strengthens the validity of the initial hit. On the other hand, as none of the M3 derivatives showed binding to MDM2, it may have been a screening artifact rather than a specific binding event.

### 2.4. De Novo Chemical Synthesis of New Molecules

**Figure 4:**
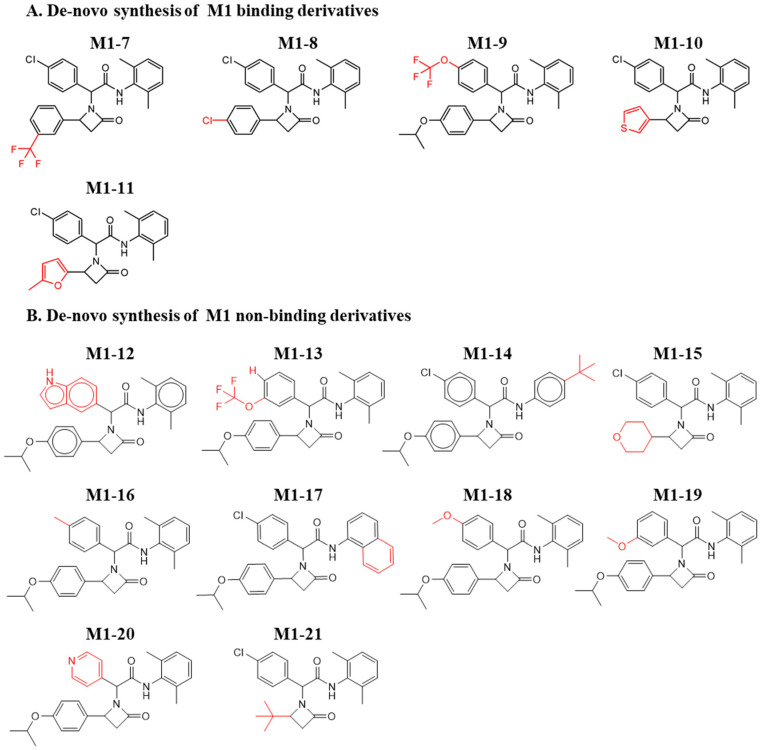
Chemical structures of de novo-synthesized M1-derived compounds. Derivatives of M1 that were synthesized and evaluated for their binding to MDM2. (**A**) Binding and (**B**) Nonbinding molecules. The red color shows the substitutions that differ from the original hit.

Though de novo synthesis does not comprise comprehensive sampling and optimization of molecules, this process, even if limited, is essential to establishing the synthetic route and validating the activity that previously relied on available chemistry. Moreover, such a step paves the way for future rigorous chemical optimization, toward improved activity. As the M3 chemical analogues did not show any binding to MDM2, we further explored de novo chemical synthesis of only M1 and M2 molecules. To this end, we designed the synthesis of several new derivatives based on the characteristics of the MDM2/p53 interaction site. This effort included adding hydrophobic groups, adding charged or hydrophilic groups directed towards the outer solvent, and adding groups capable of fulfilling specific interactions with sidechains lining the binding site. Notably, the addition of the hydrophobic groups was aimed at increasing shape complementarity towards the MDM2 binding site; and the addition of the charged or hydrophilic groups was aimed at improving solubility and stability. Figure 4A shows the de novo-synthesized M1 derivatives with binding to MDM2. The newly synthesized molecules retained central oxoazetidine acetamide, in which the original chlorophenyl and the isopropoxyphenyl were substituted with alternative chemical groups. Molecules M1-7 to M1-11 show binding to MDM2. Figure 4B shows de novo-synthesized derivatives that did not retain binding. In M1-12, chlorobenzene was replaced by an indole; in M1-9, trifluoromethoxy in the para position retained its activity. However, this in contrast to meta in M1-13 with no binding, indicating sensitivity in substituting this position. This is congruent to the inactivity of M1-18 and M1-19, for which no binding was observed with methoxy in the para and meta positions. Moreover, substitution with the large tert-butyl in M1-14, as well as with the remaining derivatives, hampered binding of the new compounds to MDM2. Figures S5 and S6 show the ITC curves of the binding and nonbinding M1 molecules, respectively.

**Figure 5:**
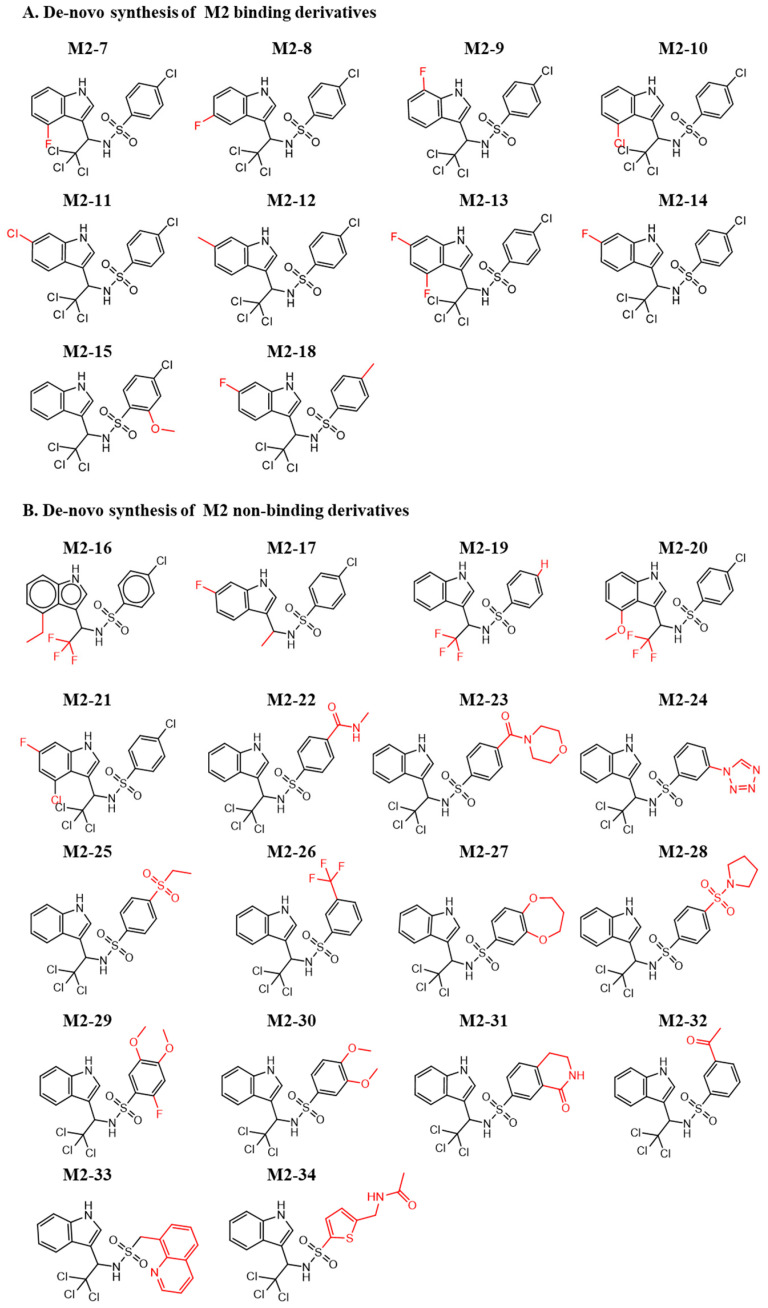
Chemical structures of de novo-synthesized M2-derived compounds. Derivatives of M2 that were synthesized and evaluated for their binding to MDM2. (**A**) Binding and (**B**) Nonbinding molecules. The red color shows the substitutions that differ from the original hit.

In a similar approach, we designed de novo-synthesized M2 derivatives. Figure 5A,B shows the synthesized M2 derivatives. In most cases, alternating the fluorine and chlorine groups on the indole retained the binding to MDM2. On the other side of the molecule, the chlorobenzene found in M2 generally did not retain its activity. An exception was compound M2-18, which harbors a methyl substitution. The substitution of the trichloro group to trifluoro (M2-16, M2-19, M2-20), with and without an additional substitution, resulted in a non-active molecule. Moreover, substitution of trichloro to methyl (M2-17) did not preserve binding to MDM2. This suggests the importance of this position for further and more precise chemical optimization. Figures S7 and S8 show the corresponding ITC curves of the binding and nonbinding molecules, respectively.

### 2.5. Cellular Activity and Viability

**Figure 6:**
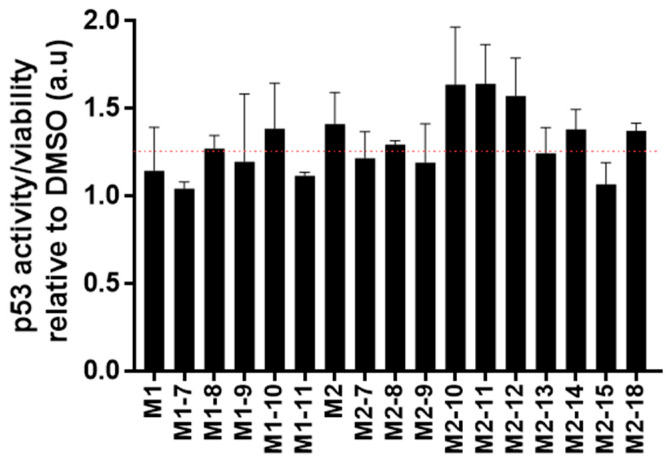
Cellular activity. A bar plot showing the fold activity of p53 in MCF7 cells treated with 15 μM of the molecules. The red line marks a 25% increase in activity.

In our next step, we embarked on evaluating the cellular activity of selected chemical structures. To this end, two functional assays were tested and analyzed, activation of p53 and cellular viability. To evaluate p53 activation, we used luciferase reporter gene, which is a robust method to evaluate p53 activity [45,46,47,48,49]. To this end, we evaluated the effect of the molecules in MCF7 cells. The latter contained wild-type p53. Cells were transfected with a plasmid that contained a firefly luciferase gene regulated by a p53 promoter. The luminescence signal level was then correlated with p53 activity. Figure 5 shows the p53 activity/viability of cells treated with the various compounds relative to cells treated with DMSO (vehicle of the compounds stock). Given that the direct binding affinity of the molecules is on the single micromolar level and that often cellular activity requires a higher level of concentration, a single dose of 15 μM was evaluated. Compounds M2-10, M2-11, and M2-12 imparted the highest signal increase, more than 60%, in p53 activity. Important to note is that additional factors such as cellular penetration and stability may affect the compound’s activity. In addition, differences in activity between similar molecules are not significant for selection of a single lead compound. As luminescence values also correlate to the number of viable cells, in addition to Figure 6, bar plots of the molecules’ effects on p53 activity and viability are shown in Figure S9. Table 1 summarizes the chemical name, structure (SMILES), and activity data of the molecules.

## 3. Discussion

Inhibition of the interaction of p53 with MDM2 exemplifies the growing interest in the therapeutic potential of modulating PPIs [50,51]. The estimated number of PPIs is ~10^6^. This, together with their relevance to a range of diseases, poses them as an attractive therapeutic target. Indeed, the past decade has witnessed the emergence of a plethora of PPI inhibitors. Among them, a large number of MDM2 inhibitors in the form of small molecules and cyclopeptides have been developed. However, although various modulators have reached clinical trials, no candidate has crossed the final line. The high potential of MDM2 inhibitors as therapeutic agents should prompt the exploration of additional molecular scaffolds that reveal enhanced inhibitory activity towards MDM2.

The diverse published MDM2 co-structures support the application of in silico screening that relies on pharmacophore constraints. Herein, based on the analysis of existing structures, we screened a virtual library against an averaged ensemble that represents the range of structures sampled by MDM2. As new chemistry often poses a major bottleneck in drug discovery, thus, an important aspect of such laboratory-scale screening is that all initial molecules will be available for following in vitro screening. Indeed, relying on existing chemistry assures the purchase and immediate evaluation of selected compounds. We coupled our computational screening that enabled the filtering of a large molecular library with ITC, as a secondary screening tool. Despite its low throughput, ITC can indicate direct binding and is considered a robust biophysical tool. Three molecules were identified as showing preliminary binding to MDM2. We further validated the binding of these preliminary hits by evaluating a focused set of commercially available structure-similar chemical analogues. Based on the aforementioned results, we defined two new molecular scaffolds capable of binding to MDM2. The first has a central oxoazetidine acetamide (M1) substituted with three groups of phenyl derivatives. The second is based on indole ethyl sulfonamide (M2). The binding affinity of the molecules is in the micromolar range, and rigorous chemical optimization is still needed to improve affinity, as well as other pharmacokinetic and chemical parameters that are essential for downstream drug development. The assay showing the cellular activity of the molecules relied on the luciferase enzyme that is expressed under the direct regulation of p53 protein. In addition to the correlation of the luminescence levels with the activity of p53, it was also dependent on the number of living cells expressing luciferase. Therefore, in addition to the p53 activity assay, we also evaluated cellular viability (Figure S9). For instance, molecules M1-10 and M1-11 had a similar effect on p53 activity (Figure S9B); however, given that M1-10 imparted a higher reduction in viability, a slightly higher p53 activity is suggested (Figure 6). Based on this rational, the most active molecules were M2-10, M2-11, and M2-12, showing an overall increase of more than 50%. The molecules have a central sulfonamide with a molecular weight lower than 500 Da. The normalized activity of the three molecules is of a similar level, and it is not feasible to determine the structure–function difference that is imparted by each of the derivatives. Moreover, additional factors are often correlated with the different cellular activity of structure-similar molecules. For instance, the chemical scaffolds of both M1 and M2 are chiral. The M1 molecule is a diastereomer, and M2 is an enantiomer. Given that not all stereoisomers are biologically active, efficient protocols for chiral separation or for pure chemistry synthesis of the active form could also contribute to increased activity. In addition, cellular activity is a multifactorial process that also depends on cell penetration, molecular stability, solubility, or chemical alteration of the molecules within the cell. These factors must be further evaluated as part of the chemical optimization to conclude a rigorous structure–function analysis. Together, our data validate the direct binding and biological activity of new chemical entities against MDM2.

## 4. Materials and Methods

### 4.1. Chemistry

All molecules were purchased from Enamine Ltd. or Akos GmbH. Chemical synthesis was performed by custom request from Enamine Ltd. All compounds were purified to 95% purity following synthesis. ^1^H 1D NMR spectra, LCMS data, and synthetic route are shown in the supplementary information.

### 4.2. MDM2 Expression and Purification

The human MDM2 containing residues 5-125, with cleavable N-terminus His tag followed by GB1 protein and TEV recognition site, was expressed in *E. coli* BL21. Transformed bacteria grew in LB until OD (600 nm) reached 0.8. Protein expression was induced with 1 mM isopropyl β-D-1-thiogalactopyranoside (IPTG) for an additional 16 h at 25 °C. Cells were harvested, and the pellet was resuspended in a lysis buffer containing 50 mM NaPi pH 8.0, 300 mM NaCl, and 10 mM imidazole. The cellular lysate was disrupted by sonication, and the soluble fraction was separated by centrifugation at 4 °C for 30 min at 20,133× *g*. The supernatant was loaded on a 5 mL nickel chelating column. Following extensive washing, MDM2 was eluted by a similar buffer with the addition of 300 mM imidazole. About 15 mL of the eluted protein was placed in a dialysis bag with TEV enzyme against 1 L of 20 mM Tris pH 8.0, 150 mM NaCl, 1 mM dithiothreitol, and 0.5 mM EDTA. The cleaved protein was loaded again on the 5 mL nickel chelating column, and the purified MDM2 was collected in the column flow-through.

### 4.3. ITC Experiments

ITC experiments were performed on PEAQ-ITC (Malvern, UK), after dialyzing MDM2 against a phosphate buffer containing 50 mM sodium phosphate pH 6.8, 150 mM NaCl, 5% DMSO, and 1 mM TCEP. MDM2 was placed in the syringe and titrated into the small molecule placed in the ITC cell. Data were analyzed using MicroCal software.

### 4.4. p53 Activation Assay and Cellular Viability

MCF-7 cells were transfected using Lipofectamine LTX Plus reagent (Invitrogen, Waltham, MA, USA), with a plasmid containing luciferase gene under the direct regulation of p53 promoter (Agilent Technologies, Santa clara, CA, USA). The day after transfection, 40,000 cells were plated in a white, opaque 96-well plate. After 6 h, the compounds were added to the wells to a final concentration of 15 μM for an additional 24 h. The reading of luciferase activity was executed via the addition of ONE-Glo reagent (Promega E6120) that includes the luciferin substrate, resulting in a bioluminescent signal. Luminescence values were read and normalized relative to equal concentrations of DMSO (vehicle)-treated cells. The results were further normalized based on the cellular viability following the same treatment protocol. Cellular viability was evaluated by metabolic ATP quantification with CellTiter-GLO reagent (Promega G7571. Luminescence values were read via Biotek SynergyH1 plate reader.

### 4.5. Generating a 3D MDM2 Model for the Docking of Small Molecules

A representative structure based on available MDM2 structures was selected using the analyze trajectory protocol in Pipeline Pilot software by Dassault systems Biovia. Subsequently, MD simulations and energy minimization were carried out with the program CHARMM [52] to refine the model and sample potential conformers using MD simulations with explicit water and counter-ions. Weak harmonic constraints were used to limit backbone movements during the entire simulation. On the basis of the results of this analysis, we generated a single averaged model that was used for molecular docking.

### 4.6. Filtering Molecules Based on Pharmacophore Constraints

Library profiling was based on analysis of the interaction pattern between known small molecule binders and MDM2, as well as on the characteristics of the MDM2 putative binding site. The fitting procedure was initiated by rapidly filtering compounds on the basis of presumably essential molecular features. This was followed by rigid matching in the 3D space to a “crude” pharmacophore model consisting of only 3–4 required features, using the Catalyst program of Dassault systems Biovia. Reference molecules/peptides for the pharmacophore generation process were taken from the following co-crystal structures (PDB IDs): 1YCR, 1RV1, 1T4E, 4DIJ, 3JZK, 3LBK, 3LBL, and 3TU1. The process was repeated for each molecule’s conformer, to filter out molecules that did not satisfy the pharmacophore requirements. Pharmacophore hypothesis and filtering were done using the search DB algorithm in the Catalyst program (Dassault Systems Biovia) [53,54]. A list of the pharmacophoric features that were defined and spatial coordinates is provided in the supplementary information. Small molecules that fulfilled all the required features within the geometrical constraints of the pharmacophore model were then ranked by “fitting value”—a calculated score based on the distance between all features in the model and the actual position of the chemical groups in the small molecule. This step resulted in reduction of the library size to approximately 500,000 compounds.

### 4.7. Molecular Docking and Final Selection of Molecules

Molecular docking of the profiled library was done using GOLD docking software by the Cambridge Crystallographic Data Centre (CCDC) with preset genetic algorithm settings optimized for virtual screening. A postdocking scoring function was applied to rank each docking pose. Several orthogonal scoring functions that are part of the score-ligands protocol were used and normalized (see list of scoring functions in SI). A consensus score that was based on the sum of normalized scores (between 0–1) was used to select a final list of the top 20,000 molecules. These were clustered into approximately 1000 clusters, and the top 500 clusters were reviewed manually. A final list of ~130 molecules was selected for further in vitro evaluation. In addition, a cutoff value was used to filter out compounds that did not satisfy specific location constraints and also to account for putative ligand interacting groups in addition to the structure-based complementary ones.

## 5. Conclusions

The integration of computational predictions with direct binding experiments is an efficient approach for the discovery of protein modulators and particularly PPI inhibitors. Computational tools can screen large molecular libraries; however, there are challenges when dealing with systems such as PPI interfaces. Complementing computational predictions with biophysical tool such as ITC leads to an efficient screening and validation engine. Herein, we applied these tools and validated the binding of new molecular scaffolds with MDM2.

## Figures and Tables

**Table 1:** Molecule parameters.

Molecule	Kd (µM)	p53 Activity/ Viability Relative to Non-Treated Cells	SMILES
M1	2.85	1.15	CC(C)Oc1ccc(cc1)[C@@H]2CC(=O)N2[C@H](C(=O)Nc3c(C)cccc3C)c4ccc(Cl)cc4
M2	2.08	1.41	Clc1ccc(cc1)S(=O)(=O)N[C@H](c2c[nH]c3ccccc23)C(Cl)(Cl)Cl
M3	16.6		COc1ccccc1c2nnc(S[C@@H]3CCCCC3=O)n2c4ccc(Cl)cc4
M4	NB		Oc1ccccc1C(=O)c2cc(c3nc4ccccc4n3c2)S(=O)(=O)c5ccccc5
M5	NB		CCOc1ccc2ccccc2c1C(=O)N3CC(=O)Nc4ccc(C)cc4[C@H]3c5ccc(F)cc5
M6	NB		COc1ccccc1[C@@H]2[N@H+](Cc3nc4ccccc4n3C)CCc5c2[nH]c6ccccc56
M1-1	WB		O=C(NC1=C(C)C=CC=C1C)C(C2=CC=C(Cl)C=C2)N3C(C4=CC=CO4)CC3=O
M1-2	NB		O=C(NC1=C(C)C=CC=C1C)C(C2=CC=C(F)C=C2)N3C(C4=CC=CO4)CC3=O
M1-3	NB		O=C(NC1=C(C)C=CC=C1C)C(C2=CC=C(OCO3)C3=C2)N4C(C5=CC=C(OC)C(OC)=C5)CC4=O
M1-4	NB		CC1=C(NC(C(C2=CC=C(F)C=C2)N3C(CC3)=O)=O)C(C)=CC=C1
M1-5	NB		O=C(C(C1)(C2=CC=C(OC)C=C2)N(C3=CC=C(Cl)C=C3)C1=O)NC4=C(C)C=CC=C4C
M1-6	WB		COc1ccc(cc1)N(C(C(=O)Nc1c(C)cccc1C)c1ccc(cc1)Cl)C=O
M1-7	33.5	1.04	CC=1C=CC=C(C)C1NC(=O)C(N2C(CC2=O)C=3C=CC=C(C3)C(F)(F)F)C=4C=CC(Cl)=CC4
M1-8	WB	1.35	CC=1C=CC=C(C)C1NC(=O)C(N2C(CC2=O)C=3C=CC(Cl)=CC3)C=4C=CC(Cl)=CC4
M1-9	WB	1.20	CC(C)OC=1C=CC(=CC1)C2CC(=O)N2C(C(=O)NC=3C(C)=CC=CC3C)C=4C=CC(OC(F)(F)F)=CC4
M1-10	19.5	1.39	CC=1C=CC=C(C)C1NC(=O)C(N2C(CC2=O)C=3C=CSC3)C=4C=CC(Cl)=CC4
M1-11	13.1	1.12	CC1=CC=C(O1)C2CC(=O)N2C(C(=O)NC=3C(C)=CC=CC3C)C=4C=CC(Cl)=CC4
M1-12	NB		CC(C)Oc1ccc(cc1)[C@H]2CC(=O)N2[C@@H](C(=O)Nc3c(C)cccc3C)c4ccc5[nH]ccc5c4
M1-13	NB		CC(C)OC=1C=CC(=CC1)C2CC(=O)N2C(C(=O)NC=3C(C)=CC=CC3C)C=4C=CC=C(OC(F)(F)F)C4
M1-14	NB		CC(C)Oc1ccc(cc1)[C@H]2CC(=O)N2[C@@H](C(=O)Nc3ccc(cc3)C(C)(C)C)c4ccc(Cl)cc4
M1-15	NB		CC=1C=CC=C(C)C1NC(=O)C(N2C(CC2=O)C3CCOCC3)C=4C=CC(Cl)=CC4
M1-16	NB		CC(C)OC=1C=CC(=CC1)C2CC(=O)N2C(C(=O)NC=3C(C)=CC=CC3C)C=4C=CC(C)=CC4
M1-17	NB		CC(C)OC=1C=CC(=CC1)C2CC(=O)N2C(C(=O)NC=3C=CC=C4C=CC=CC34)C=5C=CC(Cl)=CC5
M1-18	NB		COC=1C=CC(=CC1)C(N2C(CC2=O)C=3C=CC(OC(C)C)=CC3)C(=O)NC=4C(C)=CC=CC4C
M1-19	NB		COC=1C=CC=C(C1)C(N2C(CC2=O)C=3C=CC(OC(C)C)=CC3)C(=O)NC=4C(C)=CC=CC4C
M1-20	NB		CC(C)OC=1C=CC(=CC1)C2CC(=O)N2C(C(=O)NC=3C(C)=CC=CC3C)C=4C=CN=CC4
M1-21	NB		CC=1C=CC=C(C)C1NC(=O)C(N2C(CC2=O)C(C)(C)C)C=3C=CC(Cl)=CC3
M2-1	NB		O=S(C1=CC=CC=C1)(NC(C2=C(C)NC3=C2C=CC=C3)C(Cl)(Cl)Cl)=O
M2-2	WB		O=S(C1=CC=CC=C1)(NC(C2=CNC3=C2C=CC=C3)C(Cl)(Cl)Cl)=O
M2-3	49.7		O=S(C1=CC=C(Cl)C=C1)(NC(C2=C(C)NC3=C2C=CC=C3)C(Cl)(Cl)Cl)=O
M2-4	NB		O=S(N1C(C2=CNC3=C2C=CC=C3)C4=C(C=CC=C4)C=C1)(C5=CC=C(F)C=C5)=O
M2-5	NB		CS(=O)(=O)NCC(c1c[nH]c2c1cccc2)c1ccccc1
M2-6	NB		Fc1ccc(cc1)S(=O)(=O)NCC(c1c[nH]c2c1cccc2)c1ccccc1
M2-7	0.979	1.22	O=S(C1=CC=C(Cl)C=C1)(NC(C2=CNC3=C2C(F)=CC=C3)C(Cl)(Cl)Cl)=O
M2-8	1.58	1.30	O=S(C1=CC=C(Cl)C=C1)(NC(C2=CNC3=C2C=C(F)C=C3)C(Cl)(Cl)Cl)=O
M2-9	WB	1.19	O=S(C1=CC=C(Cl)C=C1)(NC(C2=CNC3=C2C=CC=C3F)C(Cl)(Cl)Cl)=O
M2-10	0.578	1.64	O=S(C1=CC=C(Cl)C=C1)(NC(C2=CNC3=C2C(Cl)=CC=C3)C(Cl)(Cl)Cl)=O
M2-11	0.704	1.64	O=S(C1=CC=C(Cl)C=C1)(NC(C2=CNC3=C2C=CC(Cl)=C3)C(Cl)(Cl)Cl)=O
M2-12	0.844	1.57	O=S(C1=CC=C(Cl)C=C1)(NC(C2=CNC3=C2C=CC(C)=C3)C(Cl)(Cl)Cl)=O
M2-13	0.489	1.08	FC=1C=C(F)C=2C(=CNC2C1)C(NS(=O)(=O)C=3C=CC(Cl)=CC3)C(Cl)(Cl)Cl
M2-14	1.22	1.38	O=S(C1=CC=C(Cl)C=C1)(NC(C2=CNC3=C2C=CC(F)=C3)C(Cl)(Cl)Cl)=O
M2-15	3.16	1.07	COC=1C=C(Cl)C=CC1S(=O)(=O)NC(C2=CNC=3C=CC=CC23)C(Cl)(Cl)Cl
M2-16	NB		CCc1cccc2[nH]cc([C@@H](NS(=O)(=O)c3ccc(Cl)cc3)C(F)(F)F)c12
M2-17	NB		CC(NS(=O)(=O)C=1C=CC(Cl)=CC1)C2=CNC=3C=C(F)C=CC23
M2-18	1.33	1.37	CC=1C=CC(=CC1)S(=O)(=O)NC(C2=CNC=3C=C(F)C=CC23)C(Cl)(Cl)Cl
M2-19	NB		FC(F)(F)C(NS(=O)(=O)C=1C=CC=CC1)C2=CNC=3C=CC=CC23
M2-20	NB		COC=1C=CC=C2NC=C(C(NS(=O)(=O)C=3C=CC(Cl)=CC3)C(F)(F)F)C12
M2-21	NB		FC=1C=C(Cl)C=2C(=CNC2C1)C(NS(=O)(=O)C=3C=CC(Cl)=CC3)C(Cl)(Cl)Cl
M2-22	NB		CNC(=O)C=1C=CC(=CC1)S(=O)(=O)NC(C2=CNC=3C=CC=CC23)C(Cl)(Cl)Cl
M2-23	NB		ClC(Cl)(Cl)C(NS(=O)(=O)C=1C=CC(=CC1)C(=O)N2CCOCC2)C3=CNC=4C=CC=CC34
M2-24	NB		ClC(Cl)(Cl)C(NS(=O)(=O)C=1C=CC=C(C1)N2C=NN=N2)C3=CNC=4C=CC=CC34
M2-25	NB		CCS(=O)(=O)C=1C=CC(=CC1)S(=O)(=O)NC(C2=CNC=3C=CC=CC23)C(Cl)(Cl)Cl
M2-26	NB		FC(F)(F)C=1C=CC=C(C1)S(=O)(=O)NC(C2=CNC=3C=CC=CC23)C(Cl)(Cl)Cl
M2-27	NB		ClC(Cl)(Cl)C(NS(=O)(=O)C=1C=CC=2OCCCOC2C1)C3=CNC=4C=CC=CC34
M2-28	NB		ClC(Cl)(Cl)C(NS(=O)(=O)C=1C=CC(=CC1)S(=O)(=O)N2CCCC2)C3=CNC=4C=CC=CC34
M2-29	NB		COC=1C=C(F)C(=CC1OC)S(=O)(=O)NC(C2=CNC=3C=CC=CC23)C(Cl)(Cl)Cl
M2-30	NB		COC=1C=CC(=CC1OC)S(=O)(=O)NC(C2=CNC=3C=CC=CC23)C(Cl)(Cl)Cl
M2-31	NB		ClC(Cl)(Cl)C(NS(=O)(=O)C=1C=CC=2CCNC(=O)C2C1)C3=CNC=4C=CC=CC34
M2-32	NB		CC(=O)C=1C=CC=C(C1)S(=O)(=O)NC(C2=CNC=3C=CC=CC23)C(Cl)(Cl)Cl
M2-33	NB		ClC(Cl)(Cl)C(NS(=O)(=O)CC=1C=CC=C2C=CC=NC12)C3=CNC=4C=CC=CC34
M2-34	NB		CC(=O)NCC1=CC=C(S1)S(=O)(=O)NC(C2=CNC=3C=CC=CC23)C(Cl)(Cl)Cl
M3-1	NB		O=C1C(SC2=NN=C(C3=CC=C(Cl)C=C3)N2C4=CC=CC=C4)CCCC1
M3-2	NB		O=C1OCCC1SC2=NN=C(C3=CC=CC=C3)N2C4=CC=C(Cl)C=C4
M3-3	NB		O=C1OCCC1SC2=NN=C(C3=CC=CC(OC)=C3)N2C4=CC=C(Cl)C=C4
M3-4	NB		O=C1OCCC1SC2=NN=C(C3=CC=CC=C3OC)N2C4=CC=C(Cl)C=C4
M3-5	NB		O=C1C(SC2=NN=C(C3=CC=NC=C3)N2C4=CC=C(F)C=C4)CCC1
M3-6	NB		COC1=CC=C(C2=NN=C(SCC(N3CCCC3)=O)N2C4=CC=C(Cl)C=C4)C=C1

NB—Non-Binding; WB—Weak/non-significant binding.

## Data Availability

Data is available upon request from the corresponding authors.

## Supplementary Materials

The following supporting information can be downloaded at: https://www.mdpi.com/article/10.3390/ph15060752/s1, Figure S1: An illustration of our virtual screening protocol; Figure S2: Binding evaluation of M1 structure-similar molecules; Figure S3: Binding evaluation of M2 structure-similar molecules; Figure S4: Binding evaluation of M3 structure-similar molecules; Figure S5: Binding evaluation of de-novo synthesized M1 derivatives; Figure S6: ITC titration curves of non-binding de-novo synthesized M1 derivatives; Figure S7: Binding evaluation of de-novo synthesized M2 derivatives; Figure S8: ITC titration curves of non-binding de-novo synthesized M2 derivatives; Figure S9: Cellular activity and viability; Figure S10. Pharmacophore constraints.
