# Supplementary material for: Application of In Silico Filtering and Isothermal Titration Calorimetry for the Discovery of Small Molecule Inhibitors of MDM2
Source: Pharmaceuticals (Basel). 2022 Jun 16;15(6):752. doi: 10.3390/ph15060752 (PMC9230431; doi:10.3390/ph15060752)
Supplement: Supplementary file 1 [file pharmaceuticals-15-00752-s001.zip › pharmaceuticals-1718514-supplementary.pdf]

Supplementary information

Application of in-silico filtering and isothermal titration calorimetry for the discovery of small molecule inhibitors of MDM2

Hen Alali [1,†], Itai Bloch [2,†], Irena Rapaport [2], Luisa Rodrigues [1], Inbal Sher [1], Tamar Ansbacher [1,3] and Maayan Gal [1,*]

[1] Department of Oral Biology, Goldschleger School of Dental Medicine, Faculty of Medicine, Tel Aviv University, Tel Aviv 6997801, Israel; henalali@mail.tau.ac.il (H.A.); luisarodrigues.10@gmail.com (L.R.); inbalasher87@gmail.com (I.S.); tamar.ansbacher@mail.huji.ac.il (T.A.)

[2] Migal—Galilee Research Institute, Tel-Hai Academic College, Upper Galilee 12210, Israel; itaib@migal.org.il (I.B.); irenar@migal.org.il (I.R.)

[3] Hadassah Academic College, Jerusalem 91010, Israel

* Correspondence: mayyanga@tauex.tau.ac.il

† These authors contributed equally to this work.

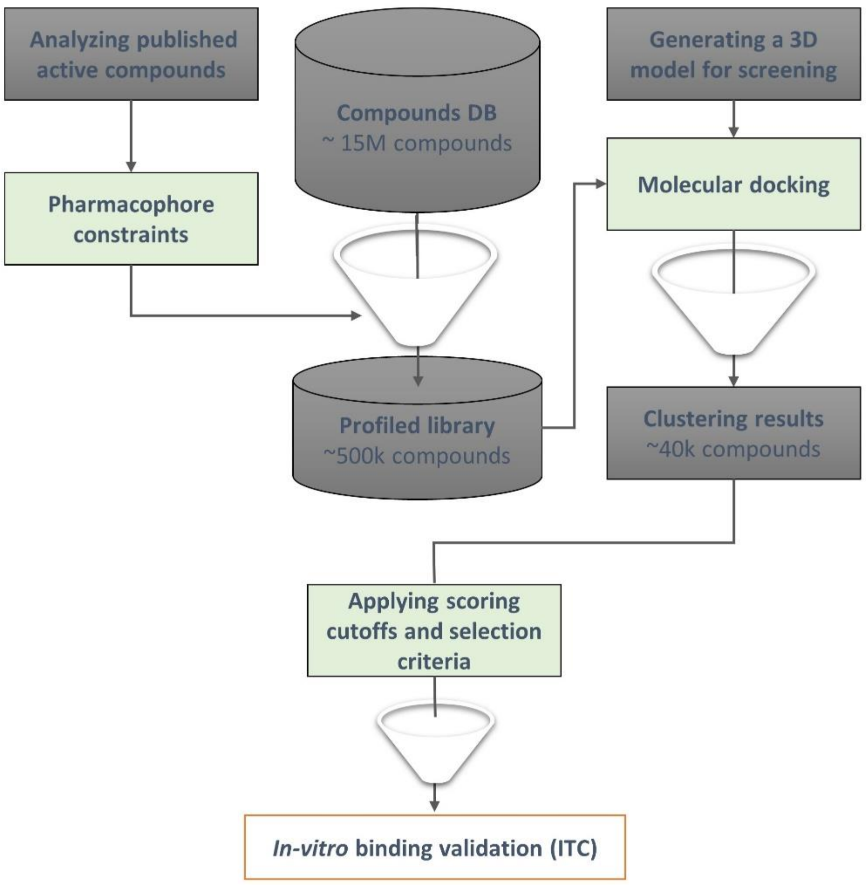

Figure S1 – Illustration of in-silico screening workflow

Figure S1. An illustration of our virtual screening protocol. Based on the defined pharmacophore constraints, initial crude filtering of the molecular library was executed. The filtered molecules were docked onto an averaged structure of MDM2. Scoring function was applied to rank the molecules, and a final set of molecules was selected based on manual analysis. Grey boxes show setup and data analysis steps. Green boxes show virtual filtering steps. The figure was adapted from Gal et al. Combinatorial Chemistry & High Throughput Screening, 2016[1].

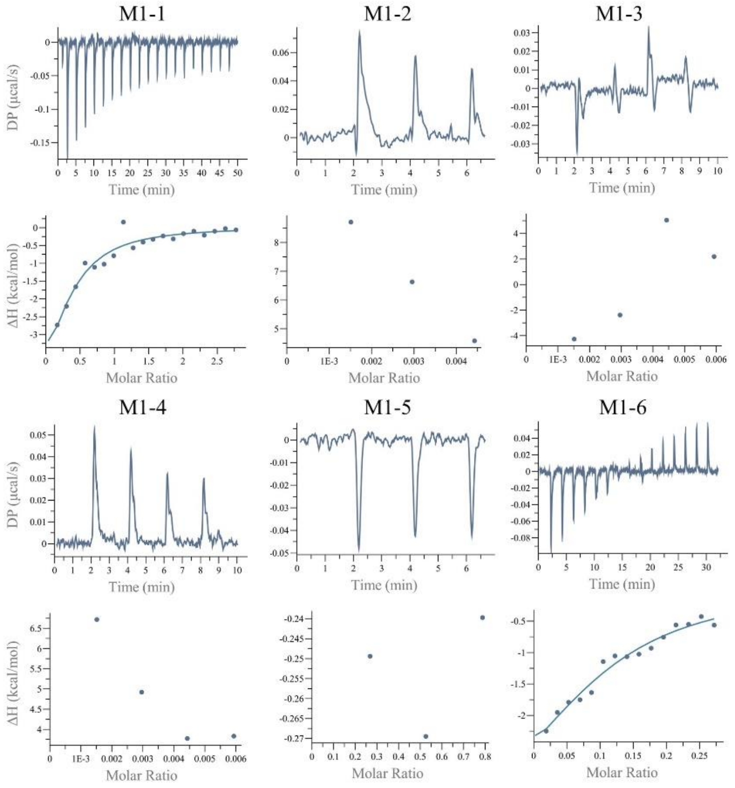

Figure S2. ITC curves that resulted from the titration of MDM2 to M1-derived molecules

Figure S2. Binding evaluation of M1 structure-similar molecules. The panels show isothermal calorimetry curves that resulted from the titration of MDM2 to the various molecules. Experiments for which no significant binding signal was observed were terminated following a limited number of injections.

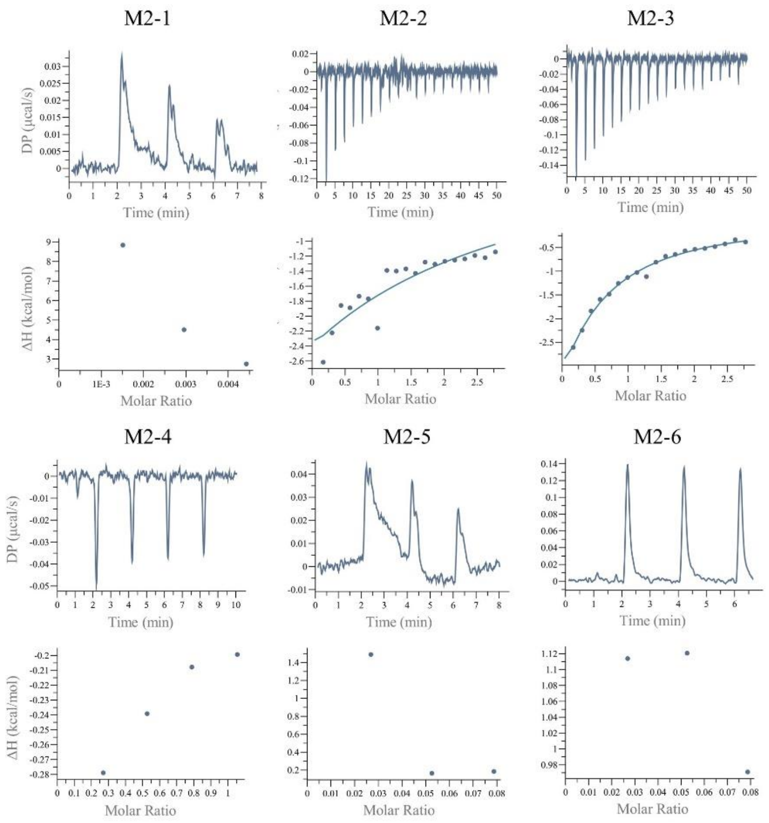

Figure S3. ITC curves that resulted from the titration of MDM2 to M2-derived molecules

Figure S3. Binding evaluation of M2 structure-similar molecules. The panels show isothermal calorimetry curves that resulted from the titration of MDM2 to the various molecules. Experiments for which no significant binding signal was observed were terminated following a limited number of injections.

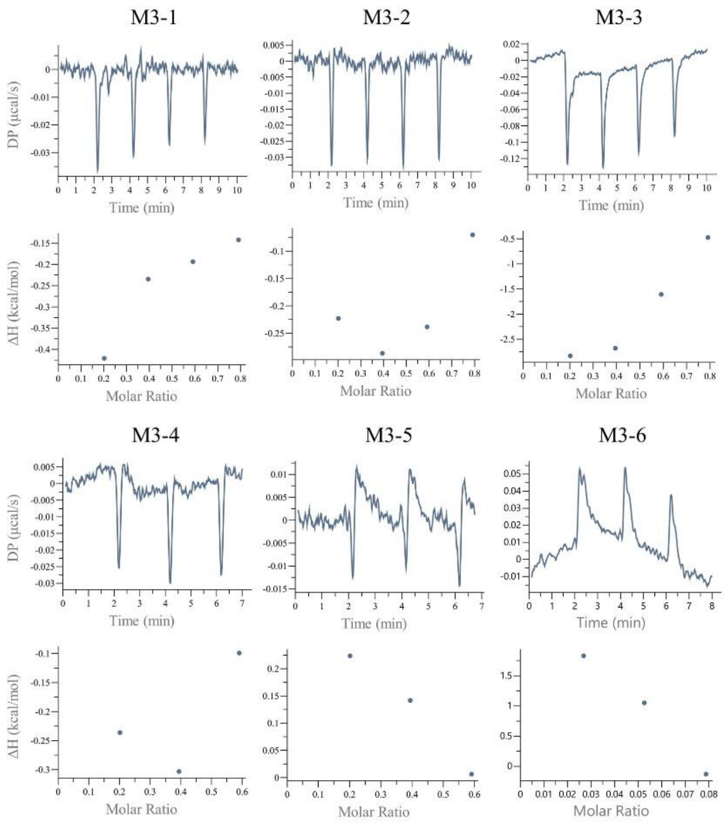

Figure S4. ITC curves that resulted from the titration of MDM2 to M3-derived molecules.

Figure S4. Binding evaluation of M3 structure-similar molecules. The panels show isothermal calorimetry curves that resulted from the titration of MDM2 to the various molecules. Experiments for which no significant binding signal was observed were terminated following a limited number of injections. No binding was observed.

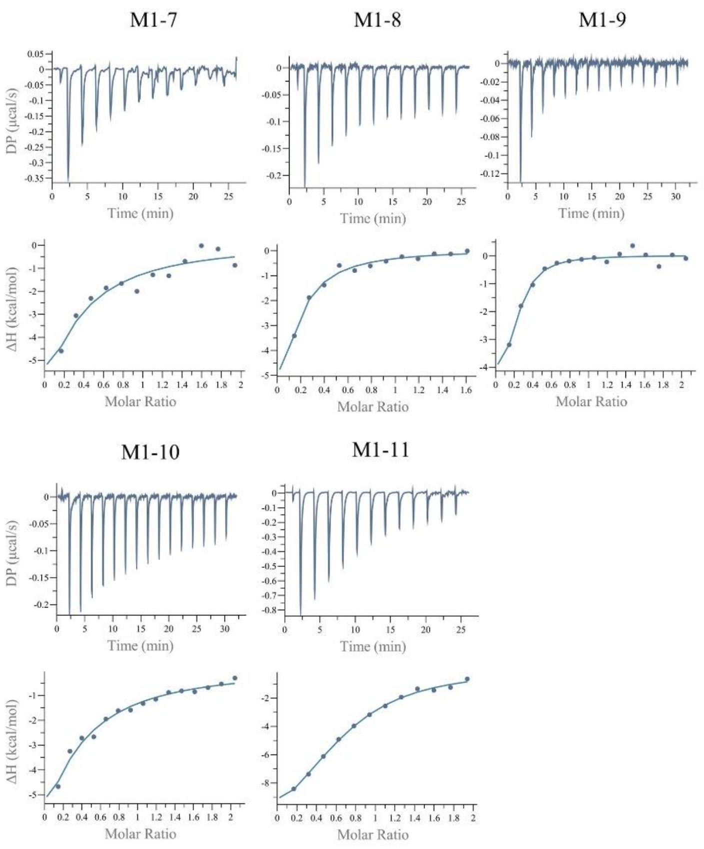

Figure S5. ITC curves that resulted from the titration of MDM2 to de-novo synthesized M1 molecules

Figure S5. Binding evaluation of de-novo synthesized M1 derivatives. The panels show isothermal calorimetry curves that resulted from the titration of MDM2 to the various molecules.

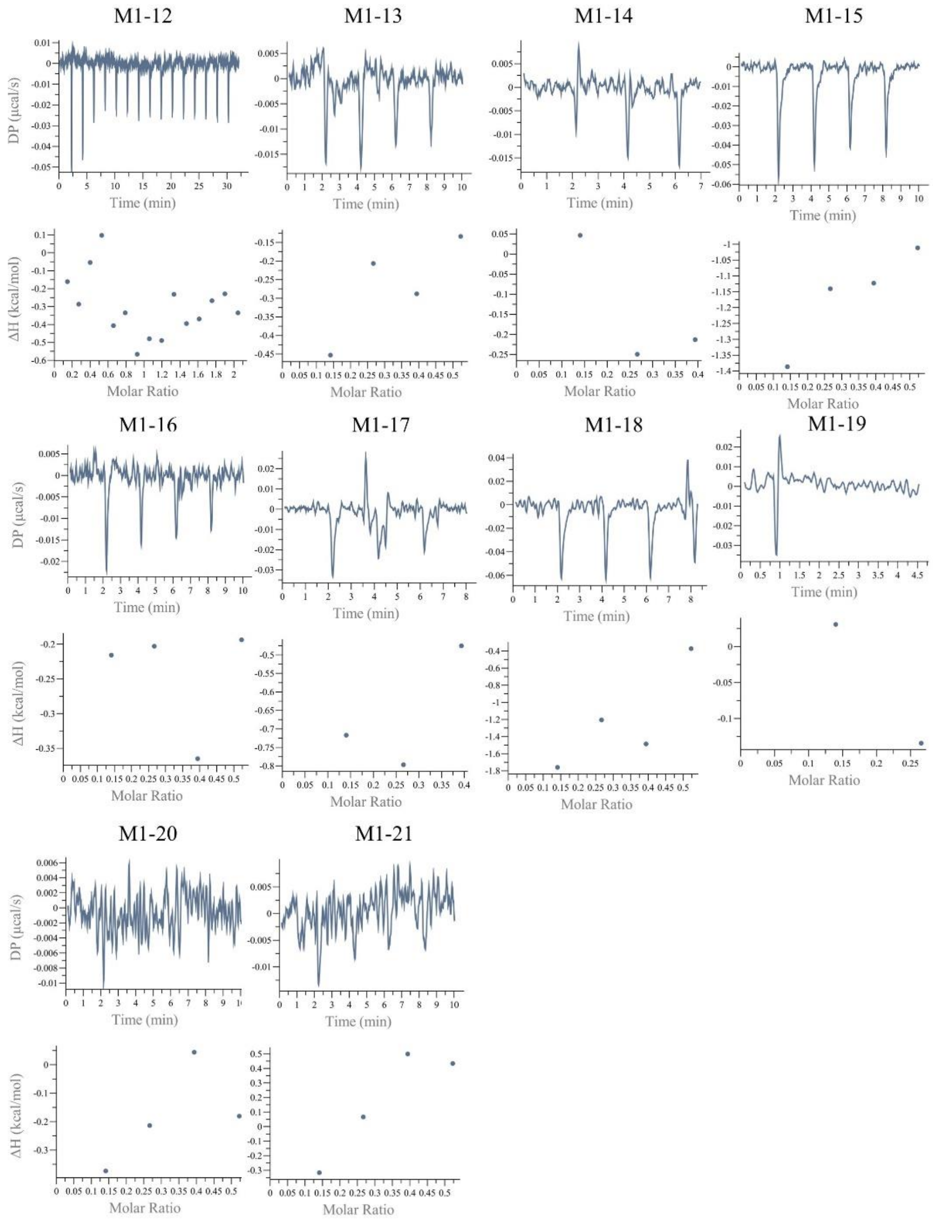

Figure S6. Non-binding ITC curves that resulted from the titration of MDM2 to de-novo synthesized M1 molecules

Figure S6. ITC titration curves of non-binding de-novo synthesized M1 derivatives. The panels show ITC curves that resulted from the titration of MDM2 to the various molecules. Experiments for which no significant binding signal was observed were terminated following a limited number of injections.

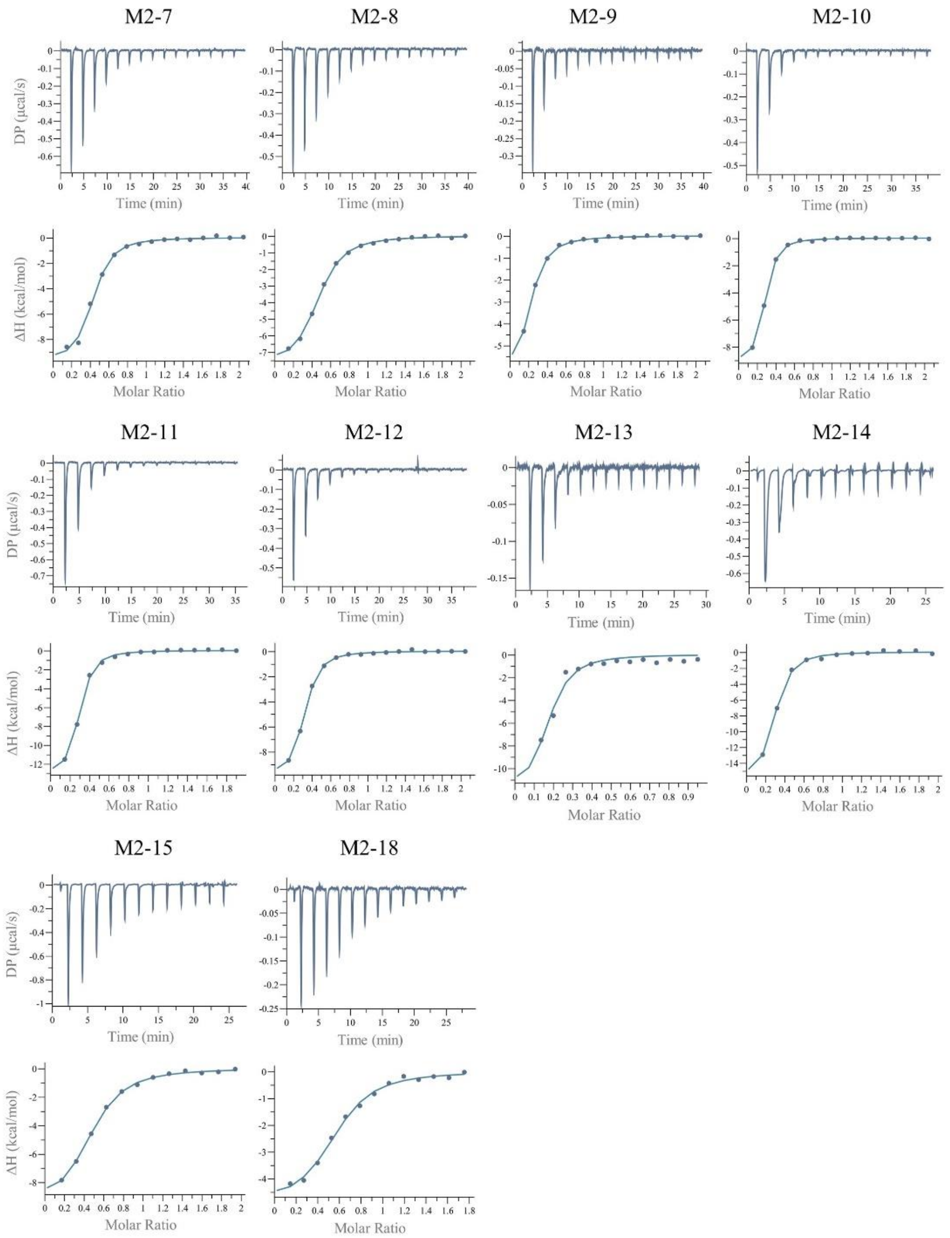

Figure S7. ITC curves that resulted from the titration of MDM2 to de-novo synthesized M2 molecules

Figure S7. Binding evaluation of de-novo synthesized M2 derivatives. The panels show isothermal calorimetry curves that resulted from the titration of MDM2 to the various molecules.

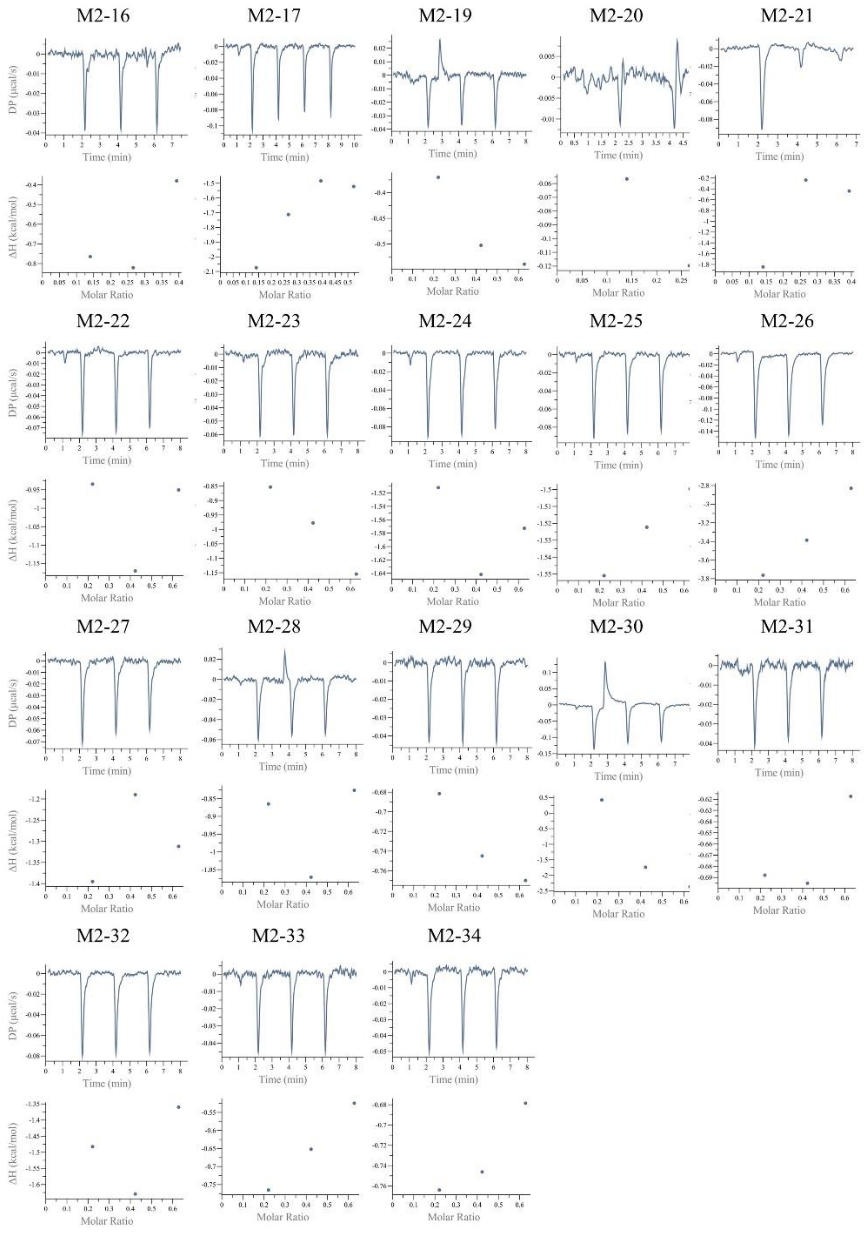

Figure S8. Non-binding ITC curves that resulted from the titration of MDM2 to de-novo synthesized M1 molecules

Figure S8. ITC titration curves of non-binding de-novo synthesized M2 derivatives. The panels show ITC curves that resulted from the titration of MDM2 to the various molecules. Experiments for which no significant binding signal was observed were terminated following a limited number of injections.

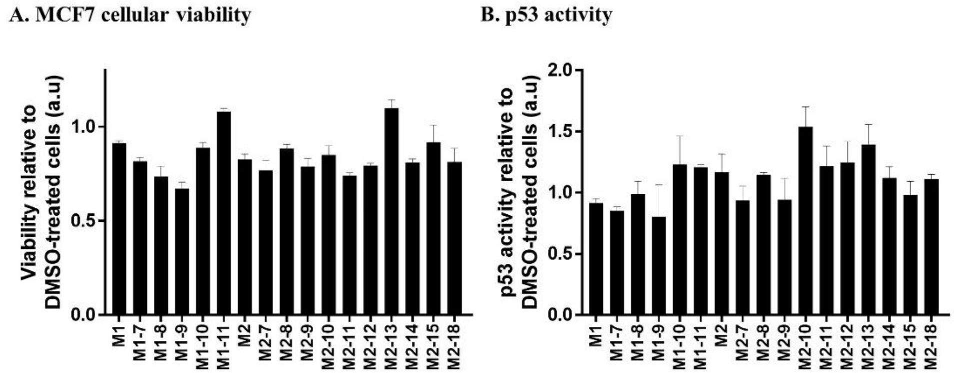

Figure S9. Evaluation of cellular viability and p53 activity following treatment with the synthesized molecules.

Figure S9. Cellular activity and viability. (A) Cellular viability. **(B)** p53 activity. Experiments were executed as described in the methods section.

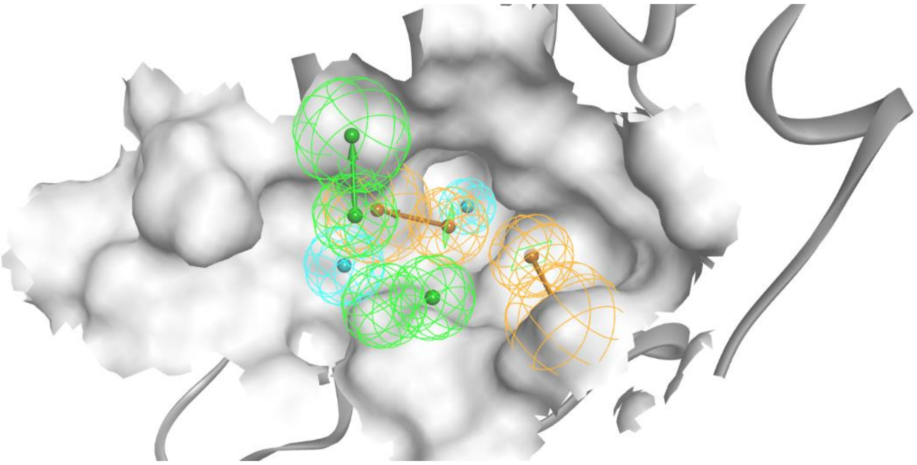

Name	X	Y	Z	Radius
HYDROPHOBIC	11.879	3.962	18.87	1.2
HB_ACCEPTOR	9.628	8.819	12.764	1.6
HB_ACCEPTOR	11.343	6.035	11.418	1.6
HB_ACCEPTOR	13.334	3.863	10.854	2.2
HYDROPHOBIC	8.44	5.337	13.597	1.6
RING_AROMATIC	14.074	9.673	16.516	1.6
RING_AROMATIC	13.654	12.6413	16.63	2.2
RING_AROMATIC	11.865	5.821	16.408	1.6
RING_AROMATIC	10.4051	3.8011	14.7381	2.2
HB_ACCEPTOR	10.981	9.472	13.824	1.6

Figure S10. Pharmacophore constraints. Hydrophobic (blue), H-acceptor (green) and aromatic region (orange) were defined. This data used to filter out molecules as further elaborated in the text.

List of scoring functions that were used for filtering and selection of molecules

- LigScore1
- PLP2
- Jain
- PMF04
- CDOCKER Score
- Goldscore
- Chemscore
- ASP

Reference

1. Gal, M.; Bloch, I.; Shechter, N.; Romanenko, O.; Shir, O. M. Efficient Isothermal Titration Calorimetry Technique Identifies Direct Interaction of Small Molecule Inhibitors with the Target Protein. *Comb. Chem. High Throughput Screen.* **2016,** 19, 4-13.

Synthetic Route

1. Molecules prepared

Index	Molecule	Structure
**1**	**M2-7**	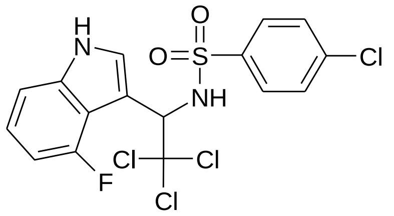
**2**	**M2-8**	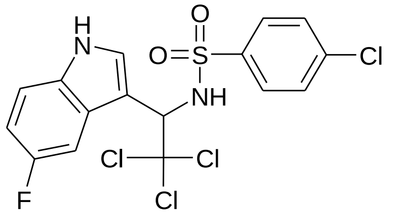
**3**	**M2-14**	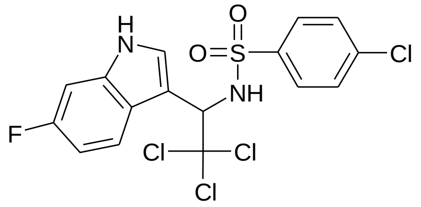
**4**	**M2-9**	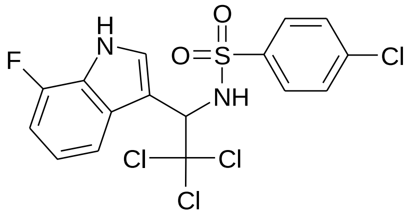
**5**	**M2-10**	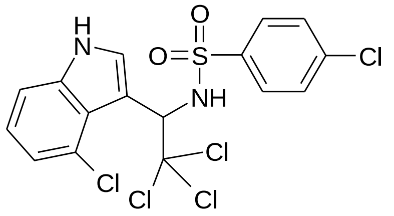
**6**	**Not in the manuscript**	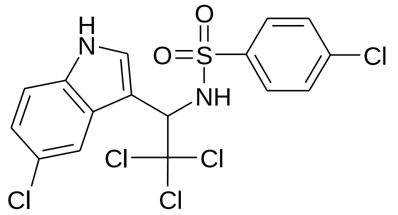
**7**	**M2-11**	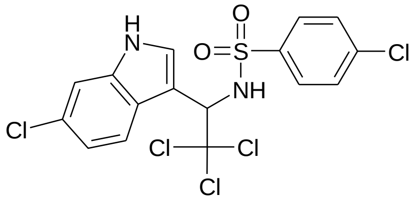

**8**	**Not in the manuscript**	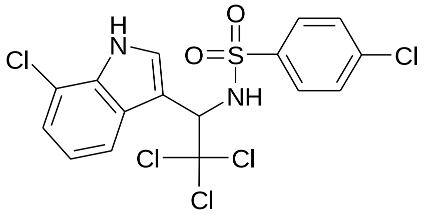
**9**	**Not in the manuscript**	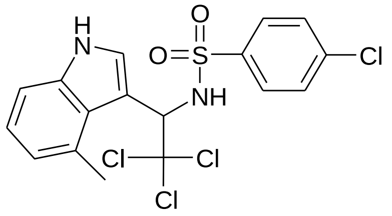
**10**	**Not in the manuscript**	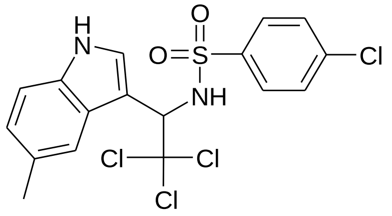
**11**	**M2-12**	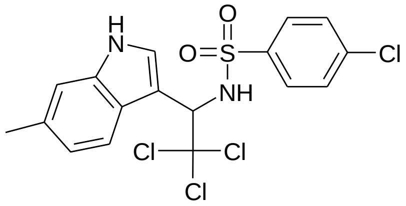
**12**	**Not in the manuscript**	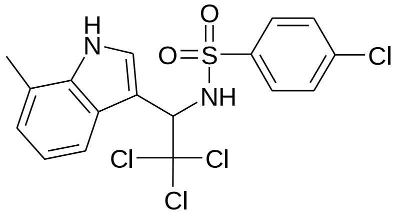

2. Synthesis

Synthesis was carried out following the general scheme given below:

Scheme 1:

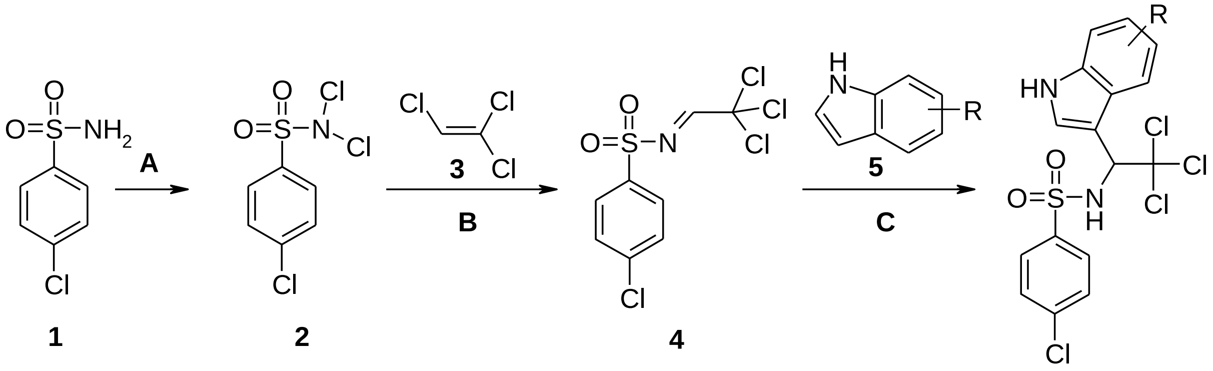

All the substituted indoles **5**, except 7-Fluoro-1H-indole, are commercially available. The synthesis of 7-Fluoro-1H-indole was performed following the scheme given below:

Scheme 2:

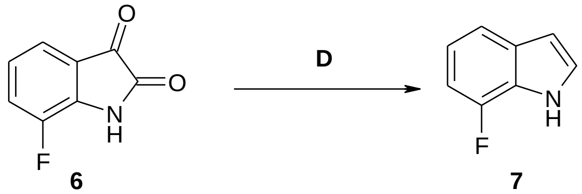

6 D 7

Step A

To a stirring solution of NaClO (65 g, 0.874 mol) in water (800 mL) 4-chloro-benzenesulfonamide (15 g, 78.5 mmol) was added and the mixture was stirred for 20 min at 20°C. Then the mixture was acidified with the acetic acid, the precipitate was filtered and dried. The solid residue was washed with hot hexane. After cooling to room temperature the product precipitated from the hexane solution. The solid was filtered and dried to afford 4.00 g (15.4 mmol, 20%) of compound **2**.

Step B.

A solution of N,N-dichloro sulfonamide 2 (5 g, 19.2 mmol) in trichloroethylene (20 mL) was refluxed in the argon atmosphere until the release of Cl_2 ceased. After the solution was cooled down to room temperature, trichloroethylene was evaporated under reduced pressure and the solid residue was recrystallized from dry benzene to afford 7.65 g (15.4 mmol, 80%) of compound **4**.

Step C. General procedure for the synthesis of molecules 1-12.

A mixture of compound **4** (1 mmol) and indole **5** (1 mmol) in dry CH_2Cl_2 (5 ml) was stirred for 16 hours at 25°C. Then CH_2Cl_2 was evaporated and the solid residue was either crystallized from IPA or purified by the column chromatography. The yields of the prepared compounds are presented in a table below.

Compound ID	Yield, %	Compound ID	Yield, %
**1**	32	**7**	5
**2**	33	**8**	33
**3**	10	**9**	27
**4**	15	**10**	44
**5**	27	**11**	13
**6**	15	**12**	60

Step D.

To a solution of **6** (31 g, 0.187 mol) in THF (300 mL) a $BH_3{\cdot}Me_2S$ complex (35.5 g, 2.5 equiv) was added dropwise at 0°C, then the mixture was stirred at rt for 16 h. After this time, the reaction was quenched by slowly adding 100 mL of demineralized water, then 2N HCl was added to pH 3. THF was removed under reduced pressure and the aqueous residue extracted with two portions of EtOAc. The organic layers were washed with two portions of brine, dried over Na_2SO_4 and evaporated under reduced pressure to give yellow oil which was then purified by silica gel chromatography yielding 10.1 g (40%, 74.8 mmol) of 7-fluoro-1H-indole **7**.

1. Molecules prepared

Index	Molecule	Structure
**1**	**M1-12**	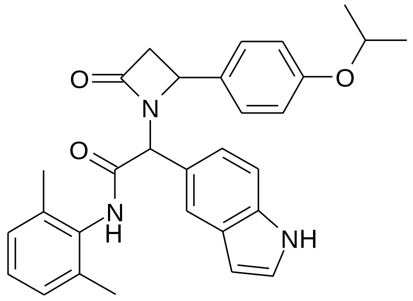
**2**	**M1-14**	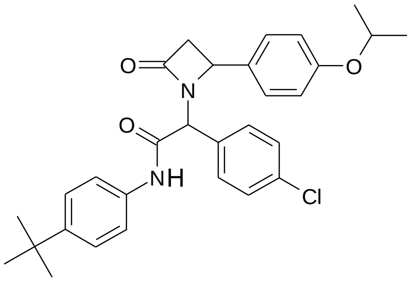
**3**	**M1-21**	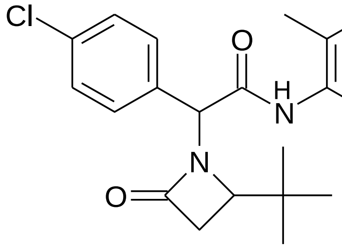
**4**	**M1-9**	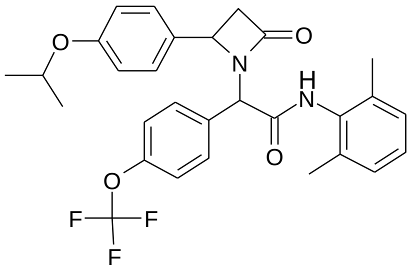
**5**	**M1-11**	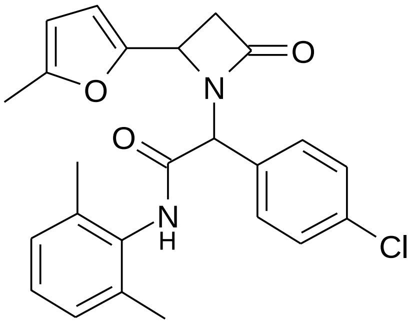

**6**	**M1-20**	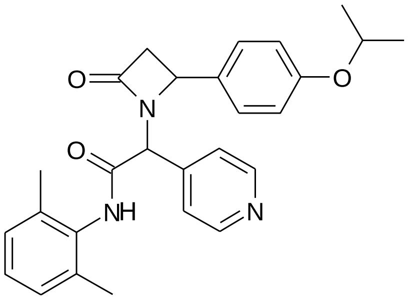
**7**	**M1-16**	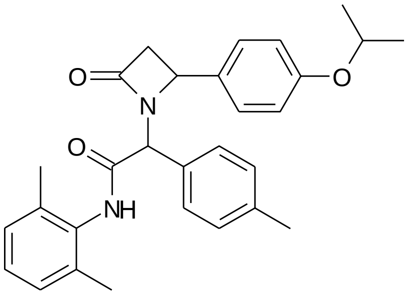
**8**	**Not in the manuscript**	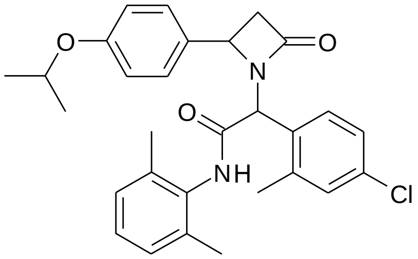
**9**	**M1-17**	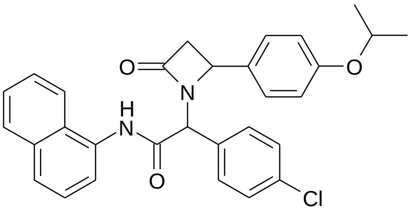
**10**	**M1-18**	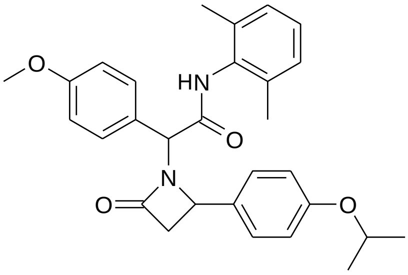
**11**	**M1-8**	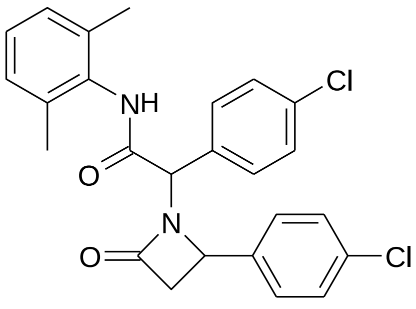

**12**	**M1-19**	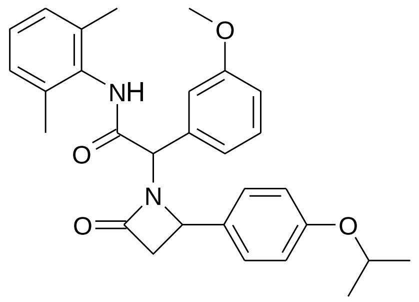
**13**	**M1-13**	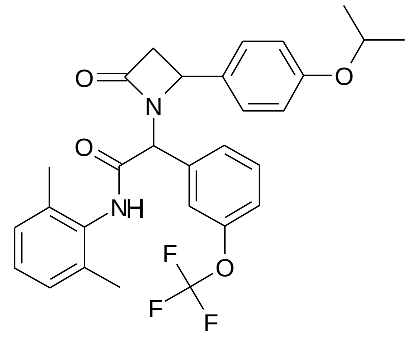
**14**	**Not in the manuscript**	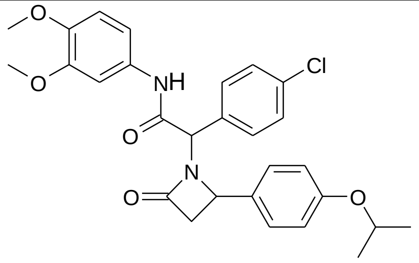
**15**	**M1-10**	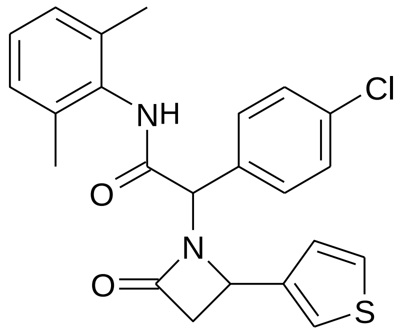
**16**	**M1-15**	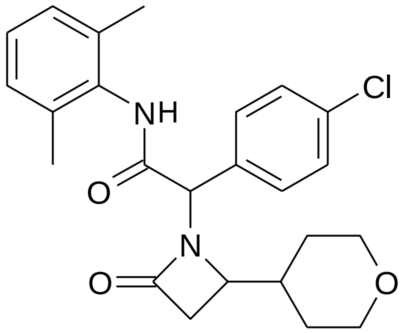

**17**	**M1-7**	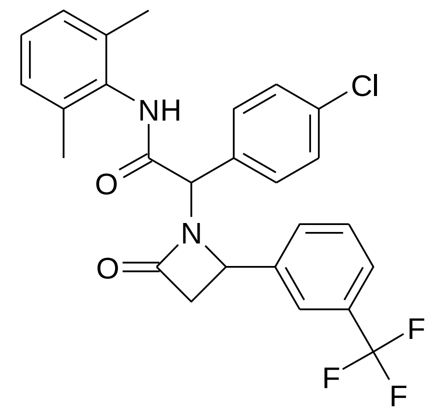

2. Synthesis

Synthesis was carried out following the general scheme given below:

Scheme 1:

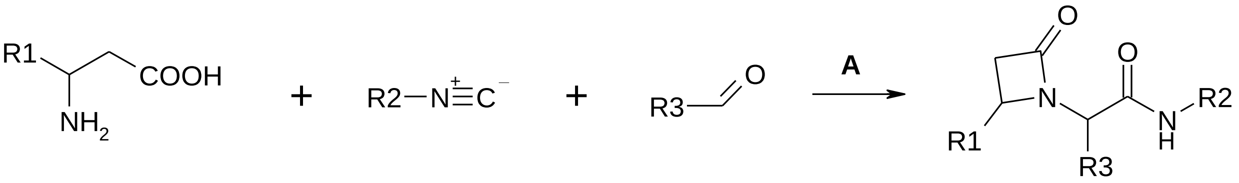

1 **2** **3** **Product**

Step A:

To a suspension of amino acid **1** (0.300 mmol) and aldehyde **3** (0.300 mmol) in dry methanol (3 mL) isonitrile **2** (0.300 mmol) was added and the mixture was stirred for 72 hours at room temperature. Then, precipitate was filtered off and washed with methanol. The filtrate was evaporated under reduced pressure to yield crude residue which purification by means of HPLC afforded target compounds with yields presented in a table below.

Compound ID	Yield, %	Compound ID	Yield, %
**17**	65	**6**	37
**16**	72	**8**	46
**15**	70	**7**	42
**14**	52	**5**	62
**13**	43	**4**	50
**11**	58	**3**	85
**12**	40	**2**	41
**10**	42	**1**	28
**9**	38		

All the amino acids except 3-amino-3-(4-isopropoxy-phenyl)-propionic acid are commercially available. The synthesis of 3-amino-3-(4-isopropoxy-phenyl)-propionic acid was performed following the scheme given below:

Scheme 2:

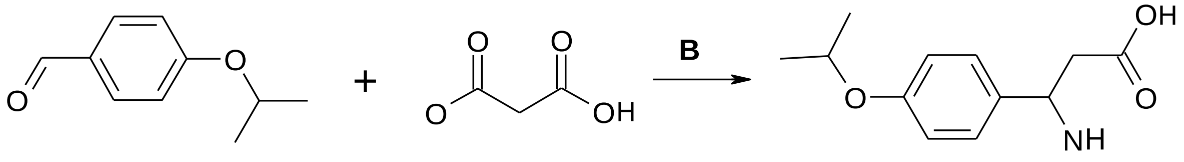

4 **5** **1a**

Step B:
A mixture of aldehyde **4** (164 g, 1000 mol), malonic acid **5** (114 g, 1096 mmol), and ammonium acetate (177 g, 2296 mmol) in 1-butanol (600 mL) was refluxed until CO_2 evolution ceased. The precipirated solid was filtered off while hot, washed in succession with boiling 1-butanol (2 x 250 mL), boiling ethanol (250 mL), water (25°C, 250 mL), and ethanol (25°C, 50 mL), and dried at 100°C to yield 133 g (596 mmol, 60%) of amino acid **1a** pure enough for the next step.

The synthesis of isocyanides **2** was performed following the general scheme given below.

Scheme 3:

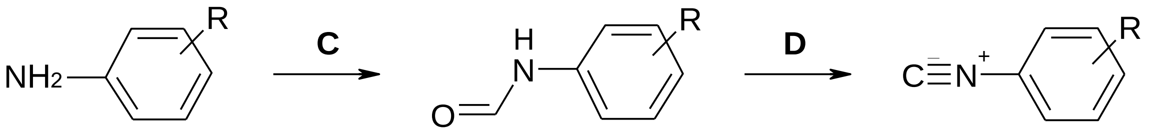

6 **7** **2**

Step C:
A solution of substituted aniline (100 mmol) and 98% formic acid (7.70 mL, 200 mmol) in toluene (60 mL) was refluxed with Dean-Stark apparatus until water release ceased. Volatiles were removed by evaporation to yield corresponding formanilide **7** in almost quantitative yield.

Step D:
To a cooled to 0°C stirring solution of formanilide **7** (10.0 mmol) and Et_3N (4.20 ml, 30 mmol) in dichloromethane (20 mL) phosphorous oxychloride (1.20 mL, 12.0 mmol) was added dropwise. After the reaction was completed, an aqueous saturated solution of sodium carbonate was added to quench the reaction at a sufficiently slow rate in order to maintain 25-30°C. After stirring for 1 hour at room temperature, more water (20 mL) and dichloromethane (10 mL) were added. The organic layer was washed with water (3 × 5 mL), dried with sodium sulfate, and evaporated. The residue was purified by column chromatography (petroleum ether/ethyl acetate = 80/1~10/1) to give isocyanobenzenes **2** with yields presented in a table below.

**Isocyanobenzene**	**Yield, %**	**Isocyanobenzene**	**Yield, %**
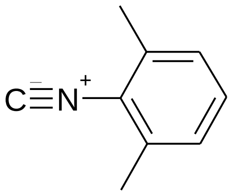	70	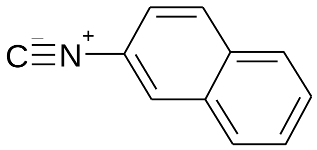	75
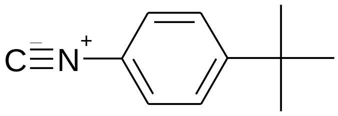	56	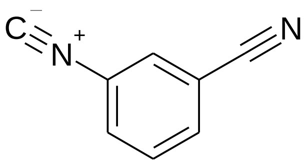	65
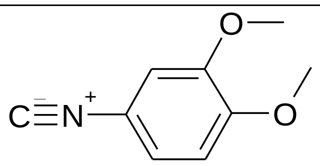	82		

1. Molecules prepared

Index	Molecule	Structure
**1**	**M2-13**	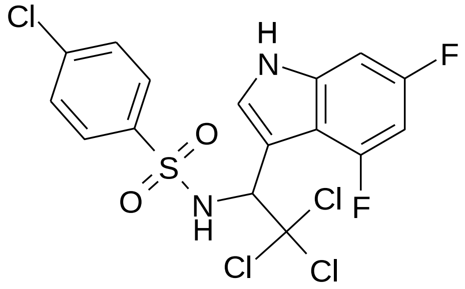
**2**	**M2-21**	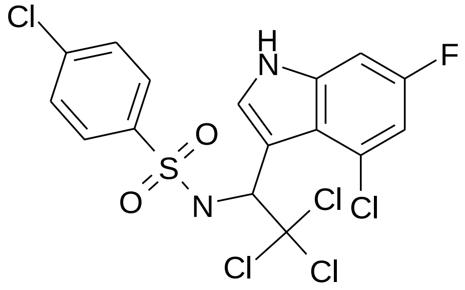
**3**	**Not in the manuscript**	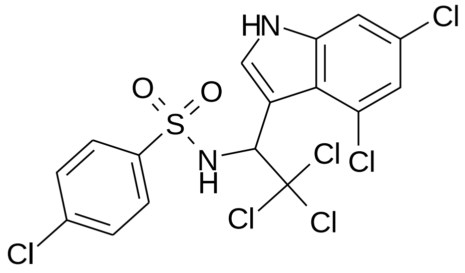
**4**	**Not in the manuscript**	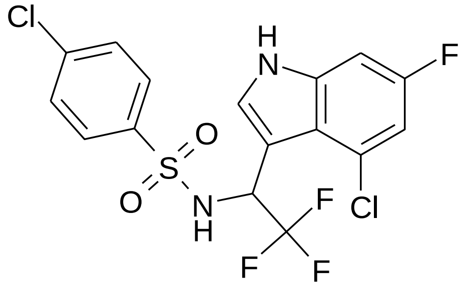
**5**	**Not in the manuscript**	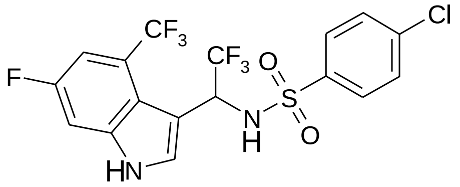
**6**	**M2-20**	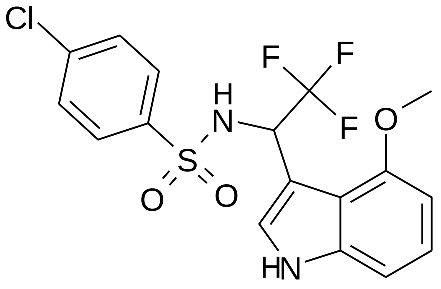

**7**	**M2-18**	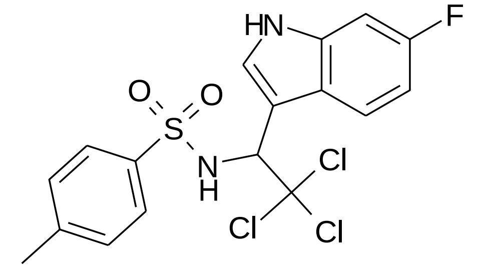
**8**	**M2-17**	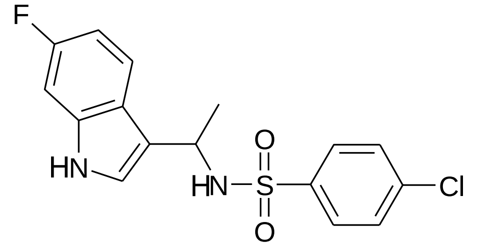
**9**	**M2-16**	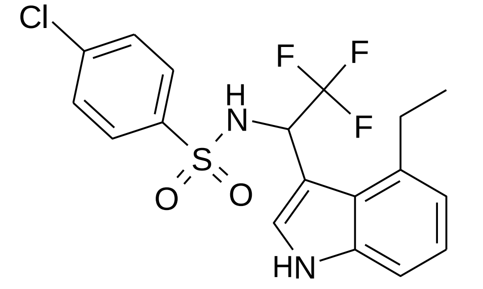

2. Synthesis

Synthesis was carried out following the scheme given below:

Scheme 1

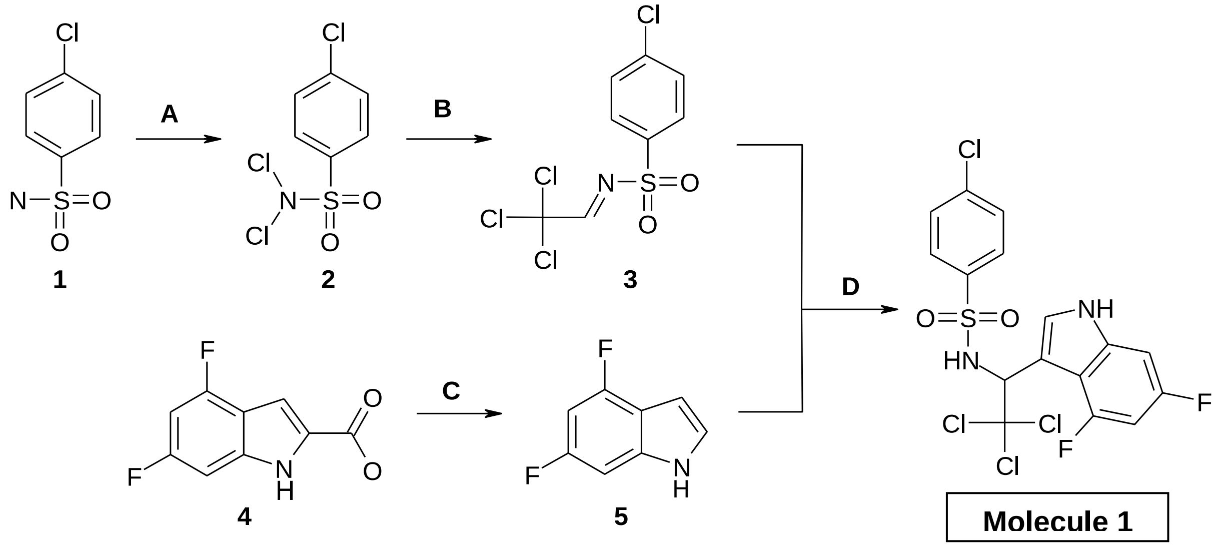

Scheme 2

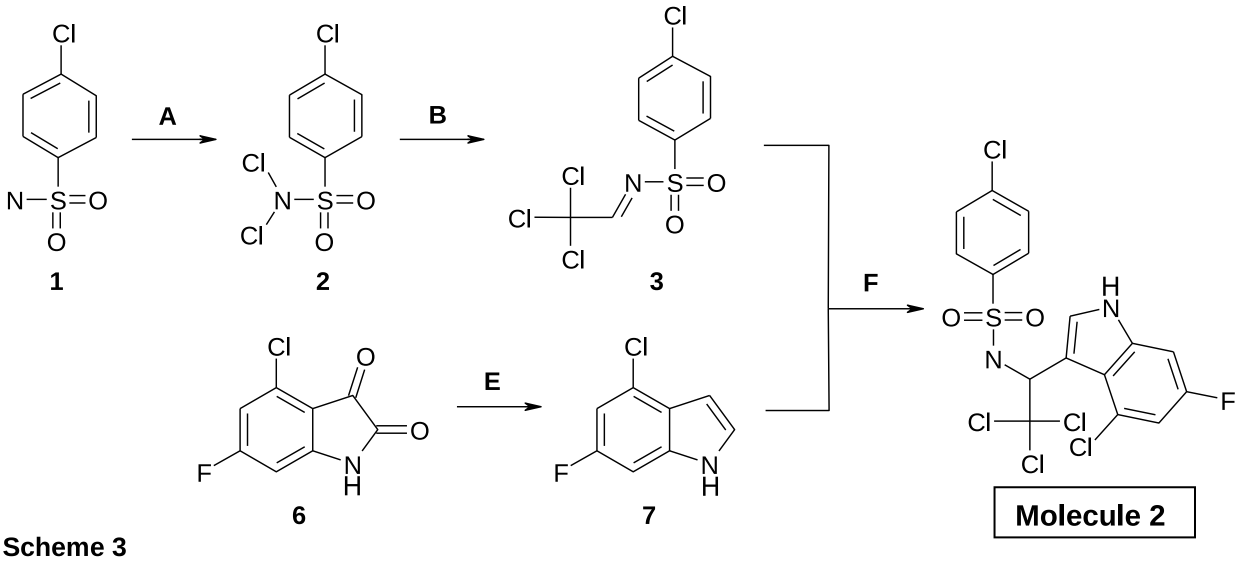

Scheme 3

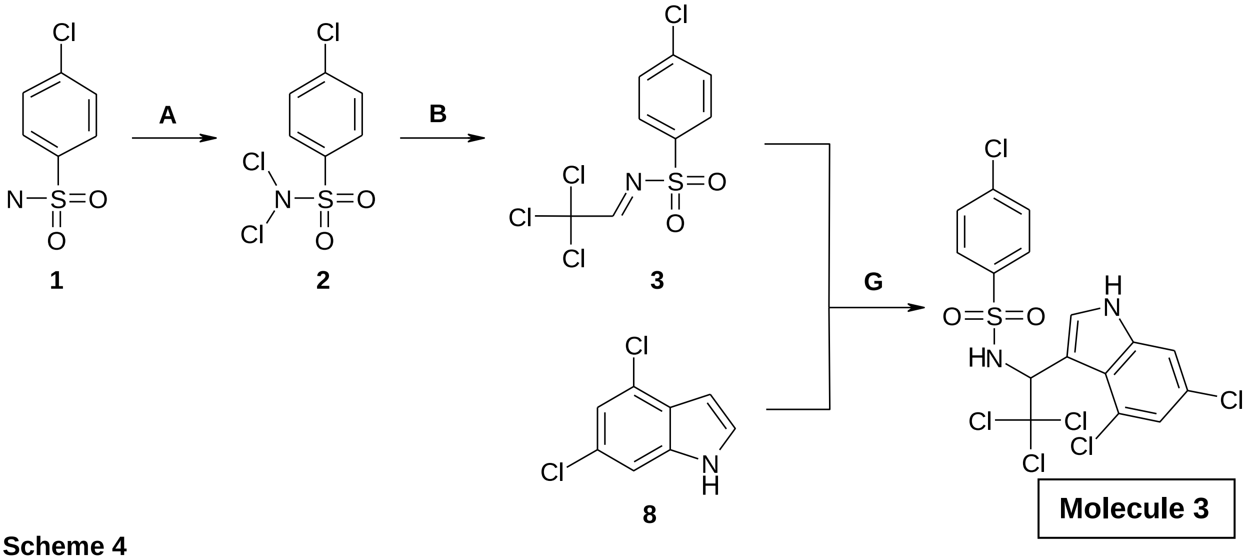

Scheme 4

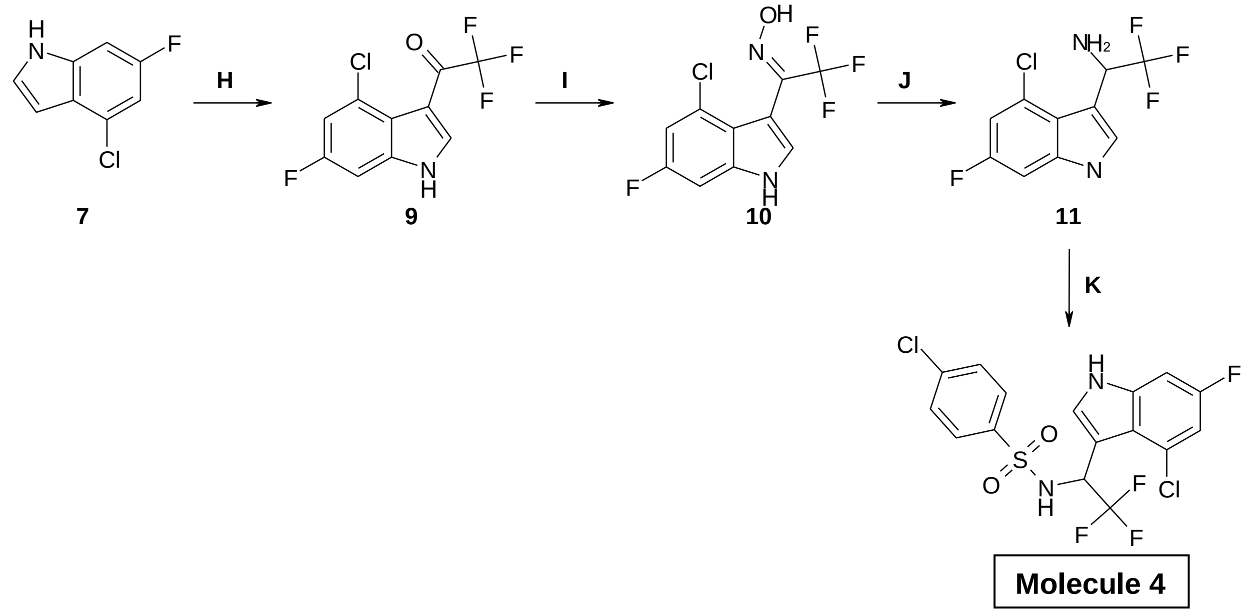

Scheme 5 (Радченко)

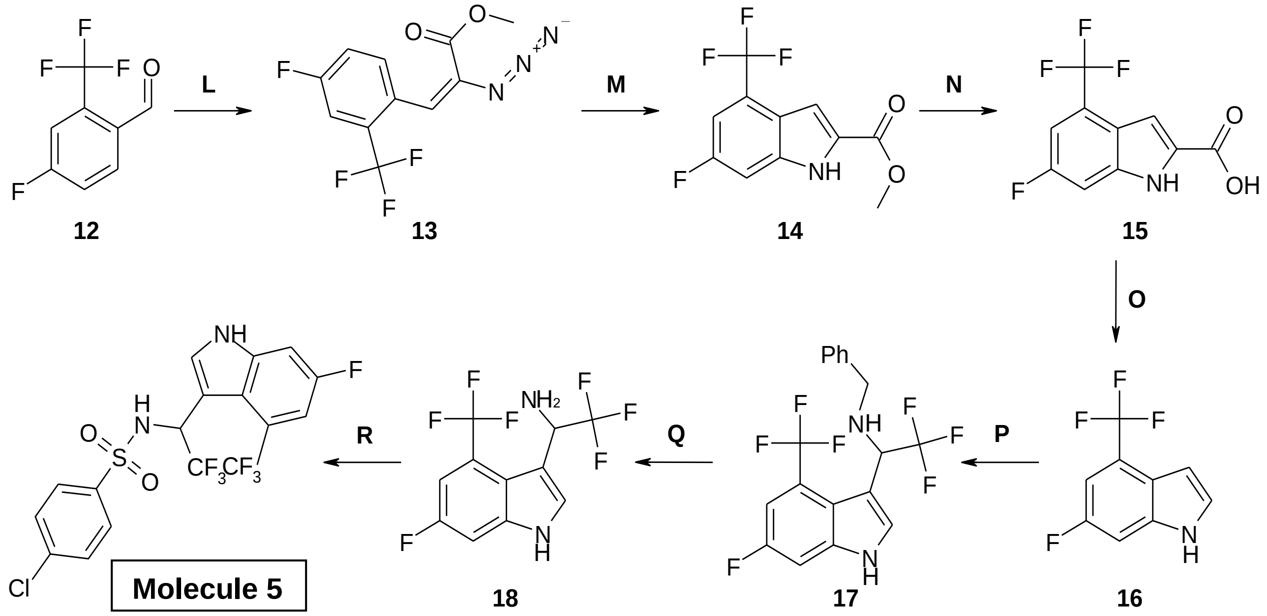

Scheme 6

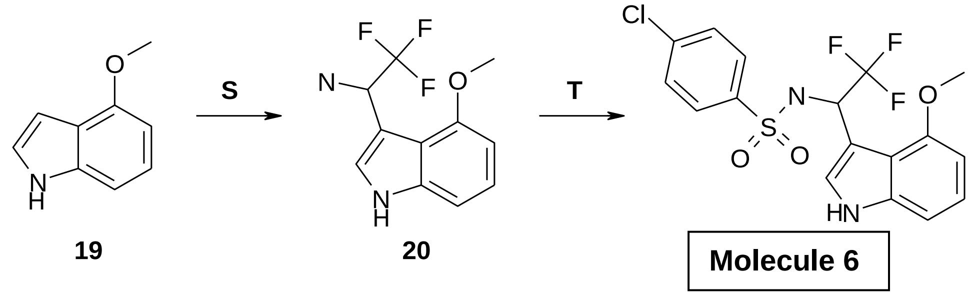

Scheme 7

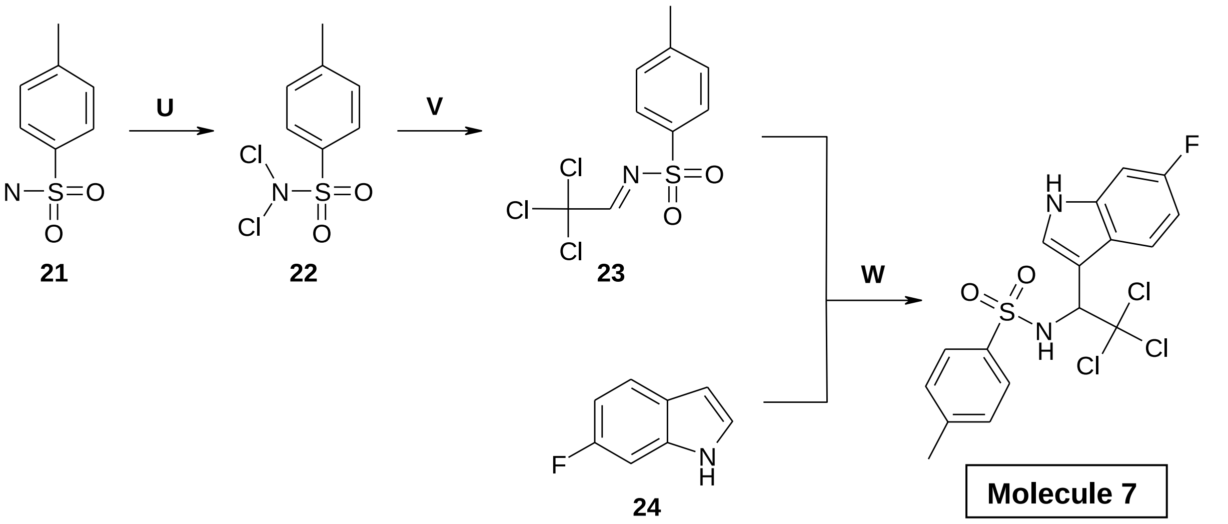

Scheme 8

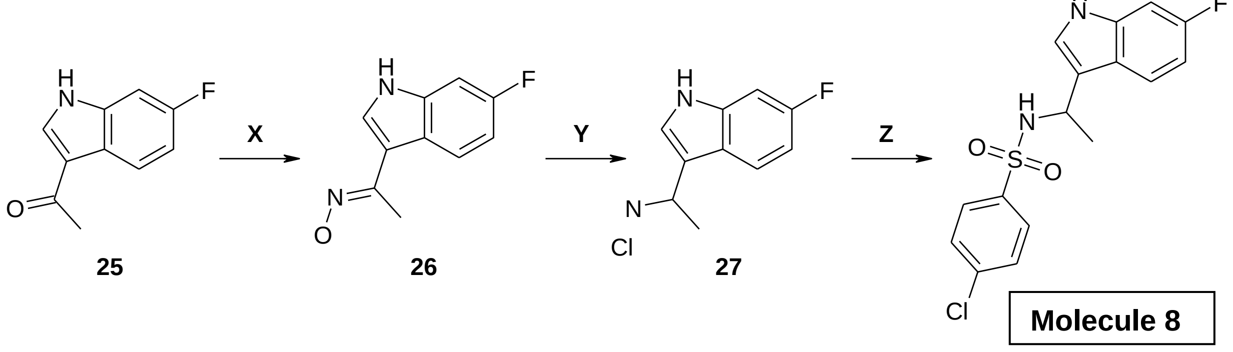

Scheme 9

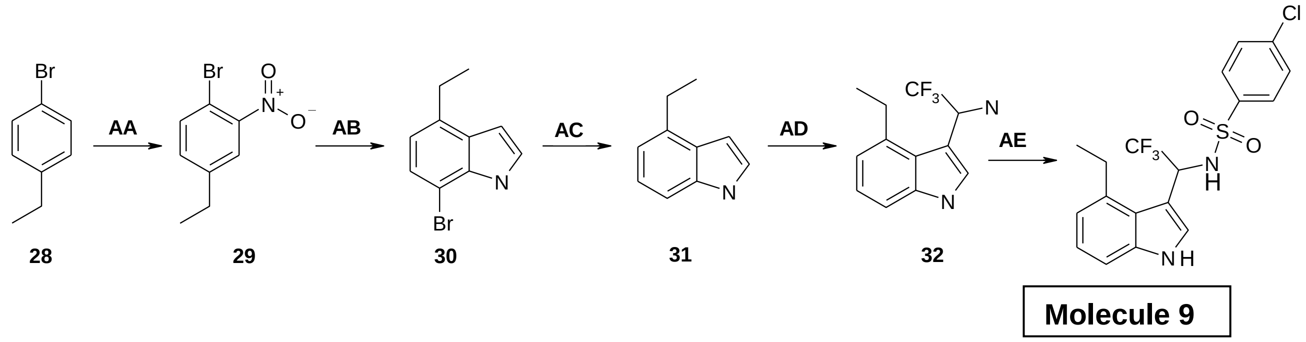

Step A:
To 37% aqueous solution of NaClO (300 mL) compound **1** (10.0 g, 52.2 mmol) was added. The mixture was stirred for 1 hour at room temperature, and then acidified to pH 4-5 with acetic acid. The precipitated solid was filtered, washed with water, dried in vacuum, and re-crystallized from hexane to yield 10.2 g (39.2 mmol, 72%) of compound **2**.

Step B:
A mixture of compound **2** (4.50 g, 17.3 mmol) and trichloroethylene (30 mL) was refluxed under argon atmosphere overnight, and then evaporated under reduced pressure. The residue was triturated with hexane/DCM mixture, filtered, and dried in vacuum to yield 2.50 g (7.79 mmol, 50%) of compound **3**.

Step C:
To a solution of compound **4** (1.30 g, 6.59 mmol) in quinoline (20 mL) CuO (0.19 g) was added and the mixture was stirred at 194°C under argon atmosphere until gas evolution ceased (~3 hours). After the mixture cooled down to room temperature, it was filtered through a small pad of silica gel, and the filtrate was diluted with EtOAc (100 mL). The mixture was washed with 6N HCl, water, dried over Na_2SO_4, and evaporated. The crude residue was purified by column chromatography to yield 0.600 g (3.92 mmol, 60%) of compound **5**.

Step D:
To a solution of compound **3** (0.503 g, 1.57 mmol) in toluene (5 mL) indole **5** (0.160 g, 1.05 mmol) and diethyl phosphite (0.036 g, 0.261 mmol) were added and the mixture was stirred overnight at room temperature. The precipitated solid was filtered, washed with hexane, and dried to obtain 0.115 g (0.243 mmol, 23%) of **molecule 1**.

Step E:
To a cooled to 0°C solution of compound **6** (7.25 g, 36.3 mmol) in dry THF (50 mL) $BH_3 \cdot Me_2S$ (4.20 g, 55.3 mmol) was added dropwise and the mixture was stirred overnight at room temperature. After completion of the reaction, it was cooled to 0°C and 5% aqueous HCl (30.0 mL) was added. The mixture was extracted with EtOAc (2 × 100 mL). The organic phase was dried over Na_2SO_4 and evaporated to give crude residue, which purification by means of column chromatography afforded 2.30 g (13.6 mmol, 37%) of compound **7**.

Step F:
To a solution of compound **3** (0.504 g, 1.57 mmol) in toluene (5 mL) indole **7** (0.346 g, 2.04 mmol) and diethyl phosphite (0.036 g, 0.261 mmol) were added and the mixture was stirred overnight at room temperature. The precipitated solid was filtered, washed with hexane, and dried to obtain 0.070 g (0.143 mmol, 7%) of **molecule 2**.

Step G:
To a solution of compound **3** (0.624 g, 1.94 mmol) in toluene (5 mL) indole **8** (0.257 g, 1.38 mmol) and diethyl phosphite (0.036 g, 0.261 mmol) were added and the mixture was stirred overnight at room temperature. The precipitated solid was filtered, washed with hexane, and dried to obtain 0.040 g (0.079 mmol, 6%) of **molecule 3**.

Step H:
To a cooled to 0°C solution of indole **7** (4.00 g, 23.6 mmol) in DMF (20 mL) TFAA was added dropwise and the mixture was stirred for 1 hour at 0°C and for 1 hour at room temperature. Then it was diluted with water and extracted with DCM. The organic phase was washed with water, dried over Na_2SO_4 and evaporated. The residue was triturated with hexane/*i*-PrOH mixture, filtered and dried to obtain 3.80 g (14.3 mmol, 60%) of compound **9**.

Step I:
A mixture of compound **9** (2.40 g, 9.04 mmol), hydroxylamine hydrochloride (6.20 g, 89.2 mmol), pyridine (7 mL), and dry ethanol (50 mL) was refluxed for 3 hours, and then evaporated. The residue was partitioned between water and MTBE. The organic layer was washed with water, brine, dried over Na_2SO_4 and evaporated to yield 1.60 g (5.70 mmol, 64%) of compound **10**.

Step J:
A mixture of compound **10** (0.900 g, 3.21 mmol), 7N ammonia solution in MeOH, and catalytical amount of Raney Ni was stirred under pressure of hydrogen (5 atm.) for 48 hours, and then filtered through a small pad of silica gel. The filtrate was evaporated to dryness under reduced pressure to obtain 0.800 g (3.00 mmol, 93%) of compound **11** pure enough for the next step.

Step K:
A mixture of amine **11** (0.056 g, 0.210 mmol), 4-chlorobenzenesulfonyl chloride (0.056 g, 0.265 mmol), and pyridine (0.5 mL) was stirred for 3 hours at room temperature, and then quenched with water. The mixture was extracted with DCM. The organic layer was washed with diluted hydrochloric acid, water, dried over Na_2SO_4 and evaporated. The residue was purified by reverse phase chromatography to obtain 0.035 g (0.079 mmol, 35%) of target **molecule 4**.

Step L:
Na (1.50 g, 65.0 mmol) was dissolved in anhydrous MeOH (80 mL), 4-fluoro-2-trifluorometylbenzaldehyde **12** (5.00 g, 26.0 mmol) was added to the solution, and the mixture was cooled to -10°C. Ethyl azidoacetate (8.38 g, 29.0 mmol) was added dropwise thereto, the mixture was stirred for 3 hours at -10°C, warmed to room temperature and treated with saturated aqueous NH_4Cl. The resulting beige solid was collected by filtration and washed with water to afford 4.00 g (13.8 mmol, 53%) of methyl 2-azido-3-(4-fluoro-2-rifluorometylphenyl)-acrylate **13**.

Step M:
A solution of azido ester **13** (4.00 g, 13.8 mmol) in xylene (200 mL) was added dropwise to refluxing xylene (250 mL) under argon atmosphere. The solution was refluxed for 1 hour, and then evaporated under reduced pressure. The resulting solid was re-crystallized using toluene to give 2.75 g (10.5 mmol, 78%) of 4-fluoro3-(trifluorometyl)-1H-indole-2-carboxylic acid methyl ester **14** a white solid.

Step N:
A suspension of 4-fluoro3-(trifluorometyl)-1H-indole-2-carboxylic acid methyl ester **14** (2.75 g, 10.5 mmol) in 2M aqueous NaOH (98 mL) was stirred at reflux for 30 minutes. After the mixture cooled down to room temperature, it was acidified and extracted with ethyl acetate (3 × 100 mL). The combined organic extracts were washed with water, dried over $MgSO_4$, and concentrated to give 2.47 g (9.99 mmol, 95%) of 4-trifluorometyl-6-fluoro-1H-indole-2-carboxylic acid **15** as a white solid.

Step O:
A mixture of 4-trifluorometyl-6-fluoro-1H-indole-2-carboxylic acid **15** (2.45 g, 9.91 mmol), copper powder (0.950 g, 15.8 mmol), and freshly distilled quinoline (30 mL) was refluxed for 2 hours. After the mixture cooled down to room temperature, it was filtered through a pad of Celite and the filtrate was poured onto crushed ice. The solution was acidified to pH 4 with concentrated

HCl, and extracted with EtOAc (3 × 100 mL). The combined extracts were washed with 2M HCl (3 × 100 mL), saturated $NaHCO_3$, brine, dried over $MgSO_4$ and concentrated. The residue was purified by silica gel chromatography (hexane/AcOEt = 85:15) to give 1.75 g (8.62 mmol, 85%) of 4-trifluorometyl-6-fluoro-1H-indole **16** as a white solid.

Step P:
To a mixture of 4-trifluorometyl-6-fluoro-1H-indole **16** (0.600 g, 2.95 mmol), trifluoroacetaldehyde methyl hemiacetal (0.468 g, 3.50 mmol), aniline (0.315 g, 3.50 mmol), and powdered 4 Å molecular sieves (0.050 g) in DCM (5 mL) $BF_3{\cdot}O(Et)_2$ (0.218 g, 0.29 mmol) was added, the resulting mixture was stirred at room temperature for 24 hours and then concentrated under reduced pressure. The residue was purified by silica gel column chromatography (hexane/EtOAc = 4:1) to give 0.678 g (1.74 mmol, 59%) of 3-(2,2,2-trifluoroacetylamino)-4-trifluorometyl-6-fluoro-1H-indole **17** as a white solid.

Step Q:
To an autoclave 10% Pt/C (0.03 g) and 3-(2,2,2-trifluoroacetyl-N-benzylamino)-4-trifluorometyl-6-fluoro-1H-indole **17** (0.650 g, 1.66 mmol) were charged. Ethanol (15 mL) was added and the mixture was purged with nitrogen (3 times) and then hydrogen (3 times). The resulting mixture was stirred under hydrogen pressure (60 psig) at 60-70°C until the uptake of hydrogen stopped (about 2 h). The resulting mixture was cooled to room temperature and filtered through Celite. The filter cake was washed with MeOH. The filtrate and rinses were concentrated in vacuo to give 0.475 g (1.58 mmol, 95%) of 3-(2,2,2-trifluoroacetyl-amino)-4-trifluorometyl-6-fluoro-1H-indole **18** as a light brown liquid.

Step R:
A 50 mL round-bottomed flask under dry nitrogen was charged with 3-(2,2,2-trifluoroacetyl-amino)-4-trifluorometyl-6-fluoro-1H-indole **18** (0.450 g, 1.50 mmol), triethylamine (0.182 g, 1.80 mmol) and dry CH_2Cl_2 (7 mL), and the mixture was cooled to 0°C. A solution of 4-chlorobenzenesulfonyl chloride (0.485 g, 2.30 mmol) in dry CH_2Cl_2 (3 mL) was added and the mixture was stirred under an atmosphere of dry nitrogen for 4 hours at room temperature. Then the solvent was removed under reduced pressure and the crude product was purified by column chromatography (EtOAc/hexane = 1:1) to give 0.285 g (0.777 mmol, 52%) of 4-chloro-N-{2,2,2-trifluoro-1-[6-fluoro-4-(trifluoromethyl)-1H-indol-3-yl]ethyl}benzene-1-sulfonamide **molecule 5** as a white solid.

Step S:
Trifluoroacetaldehyde ethyl hemiacetal (1.77 g, 12.3 mmol) was mixed with HMDS (1.98 g, 12.3 mmol) at 0°C and the mixture was the stirred for 2 hours at 50°C. After it cooled down to room temperature, a solution of compound **19** (1.80 g, 12.2 mmol) in DCM (30 mL) was added thereto and the reaction was cooled to 0°C. Boron trifluoride diethyl etherate (1.74 g, 12.3 mmol) was added dropwise and the mixture was stirred for 2 hours at 10°C. The reaction was quenched with water (30 mL), neutralized with aqueous $NaHCO_3$ solution, and extracted with EtOAc. The organic phase was dried over Na_2SO_4 and evaporated to give crude residue which purification by column chromatography afforded 0.730 g (2.99 mmol, 24%) of compound **20**.

Step T:
A mixture of amine **20** (0.175 g, 0.717 mmol), 4-chlorobenzenesulfonyl chloride (0.180 g, 0.853 mmol), and pyridine (1 mL) was stirred for 3 hours at room temperature, and then quenched with water. The precipitated solid was filtered, washed with water and water/ethanol mixture, and dried to obtain 0.275 g (0.656 mmol, 69%) of target **molecule 6**.

Step U:
To a 37% aqueous solution of NaClO (700 mL) compound **21** (21.3 g, 124 mmol) was added, the mixture was stirred for 1 hour at room temperature, and then acidified to pH 4-5 with acetic acid. The precipitated solid was filtered, washed with water, dried in vacuum, and re-crystallized from hexane to yield 28.0 g (117 mmol, 93%) of compound **22**.

Step V:

A mixture of compound **22** (4.00 g, 16.7 mmol) and trichloroethylene (30 mL) was refluxed under argon atmosphere overnight, and then evaporated under reduced pressure. The residue was triturated with hexane, filtered, and dried in vacuum to yield 4.25 g (14.1 mmol, 85%) of compound **23**.

Step W:

To a solution of compound **23** (0.690 g, 2.30 mmol) in toluene (10 mL) indole **24** (0.310 g, 2.29 mmol) and diethyl phosphite (0.063 g, 0.456 mmol) were added and the mixture was stirred overnight at room temperature. The precipitated solid was filtered, washed with hexane, and dried to obtain 0.475 g (1.09 mmol, 48%) of **molecule 7**.

Step X:

A mixture of compound **25** (4.00 g, 22.6 mmol), hydroxylamine hydrochloride (3.20 g, 46.0 mmol), sodium acetate (9.30 g, 113 mmol), and ethanol (100 mL) was refluxed for 3 hours, and then evaporated to dryness under reduced pressure. Water was added to the residue, the insoluble solid was filtered, washed with water, and dried under reduced pressure to obtain 4.00 g (20.8 mmol, 91%) of compound **26**.

Step Y:

A mixture of compound **26** (3.00 g, 15.6 mmol), 7N ammonia solution in MeOH, and catalytical amount of Raney Ni was stirred under pressure of hydrogen (5 atm.) overnight, and then filtered through a small pad of silica gel. The filtrate was evaporated to dryness under reduced pressure and the residue was dissolved in DCM. HCl was added to the solution, the precipitated solid was filtered, washed with acetone, and dried to obtain 0.800 g (3.73 mmol, 24%) of compound **27** as HCl salt.

Step Z:

A mixture of amine **27** (0.183 g, 1.03 mmol), 4-chlorobenzenesulfonyl chloride (0.233 g, 1.10 mmol), and pyridine (2 mL) was stirred for 3 hours at room temperature, and then quenched with water. The precipitated solid was filtered, washed with water and water/ethanol mixture, and dried to obtain 0.100 g (0.283 mmol, 33%) of target **molecule 8**.

Step AA:

To a cooled to 0°C stirring solution of compound **28** (30.6 g, 165 mmol) in H_2SO_4 (100 mL) HNO_3 (10.4 g) was added dropwise and the mixture was stirred for 20 minutes at room temperature. Additional portion of HNO_3 (2.00 g) was then added and the mixture was stirred for another 20 minutes. After the reaction completed, it was poured into an aqueous solution of K_2CO_3 and extracted with EtOAc. The organic phase was washed with an aqueous solution of K_2CO_3, water, brine, dried over Na_2SO_4 and evaporated to yield a mixture of isomers, which separation by means of column chromatography afforded 6.00 g (26.1 mmol, 16%) of compound **29**.

Step AB:

To a stirring solution of compound **29** (5.10 g, 22.2 mmol) in THF (70 mL) 1M THF solution of vinylmagnesium bromide (78 mL) was added under argon atmosphere maintaining inner temperature below -45°C and the mixture was stirred for 30 minutes. Saturated solution of NH_4Cl was then added and the mixture was extracted with Et_2O. The organic phase was washed with water, brine, dried over Na_2SO_4 and evaporated. The residue was purified by means of column chromatography to yield 2.40 g (10.7 mmol, 48%) of compound **30**.

Step AC:

A mixture of compound **30** (1.10 g, 4.91 mmol), methanol, TEA (0.500 g, 4.94 mmol) and 10% Pd/C (0.260 g) was stirred under hydrogen atmosphere for 2 hours at room temperature. The resulting suspension was filtered, the filtrate was concentrated under reduced pressure, and the residue was partitioned between water and DCM. The organic phase was separated, washed

with water, dried over Na_2SO_4, and evaporated to yield 0.680 g (4.69 mmol, 97%) of compound **31**.

Step AD:

Trifluoroacetaldehyde ethyl hemiacetal (0.68 g, 4.72 mmol) was mixed with HMDS (0.770 g, 4.77 mmol) at 0°C and the mixture was the stirred for 2 hours at 50°C. After it cooled down to room temperature, a solution of compound **31** (0.690 g, 4.75 mmol) in DCM (30 mL) was added thereto and the reaction was cooled to 0°C. Boron trifluoride diethyl etherate (0.670 g, 4.72 mmol) was added dropwise and the mixture was stirred for 2 hours at 10°C. The reaction was quenched with water (30 mL), neutralized with aqueous $NaHCO_3$ solution, and extracted with EtOAc. The organic phase was dried over Na_2SO_4 and evaporated to give crude residue which purification by column chromatography afforded 0.270 g (1.43 mmol, 30%) of compound **32**.

Step AE:

A mixture of amine **32** (0.270 g, 1.43 mmol), 4-chlorobenzenesulfonyl chloride (0.283 g, 1.34 mmol), and pyridine (1 mL) was stirred for 3 hours at room temperature, then quenched with water, and extracted with DCM. The organic phase was washed with 1M HCl, water, dried over Na2SO4, and evaporated. The residue was purified by column chromatography to obtain 0.120 g (0.330 mmol, 26%) of target compound **molecule 9**.
